# Research and Development of Natural Product Tanshinone I: Pharmacology, Total Synthesis, and Structure Modifications

**DOI:** 10.3389/fphar.2022.920411

**Published:** 2022-07-11

**Authors:** Xing Huang, Lili Jin, Hao Deng, Dan Wu, Qing-kun Shen, Zhe-shan Quan, Chang-hao Zhang, Hong-Yan Guo

**Affiliations:** Key Laboratory of Natural Medicines of the Changbai Mountain, Affifiliated Ministry of Education, College of Pharmacy, Yanbian University, Yanji, China

**Keywords:** tanshinone I, pharmacology, total synthesis, structure-modifications, danshen

## Abstract

*Salvia miltiorrhiza* (*S. miltiorrhiza*), which has been used for thousands of years to treat cardiovascular diseases, is a well-known Chinese medicinal plant. The fat-soluble tanshinones in *S. miltiorrhiza* are important biologically active ingredients including tanshinone I, tanshinone IIA, dihydrotanshinone, and cryptotanshinone. Tanshinone I, a natural diterpenoid quinone compound widely used in traditional Chinese medicine, has a wide range of biological effects including anti-cancer, antioxidant, neuroprotective, and anti-inflammatory activities. To further improve its potency, water solubility, and bioavailability, tanshinone I can be used as a platform for drug discovery to generate high-quality drug candidates with unique targets and enhanced drug properties. Numerous derivatives of tanshinone I have been developed and have contributed to major advances in the identification of new drugs to treat human cancers and other diseases and in the study of related molecular mechanisms. This review focuses on the structural modification, total synthesis, and pharmacology of tanshinone I. We hope that this review will help understanding the research progress in this field and provide constructive suggestions for further research on tanshinone I.

## 1 Introduction

The dry roots of the Chinese medicinal plant *Salvia miltiorrhiza* (*S. miltiorrhiza*), also known as Danshen, have been widely used in traditional Chinese medicine to treat heart and vascular diseases (Tian and Wu, 2013). *S. miltiorrhiza* has been shown to have therapeutic effects on cardiovascular and cerebrovascular diseases, including angina pectoris and myocardial infarction (Wang et al., 2007).

**FIGURE 1:**
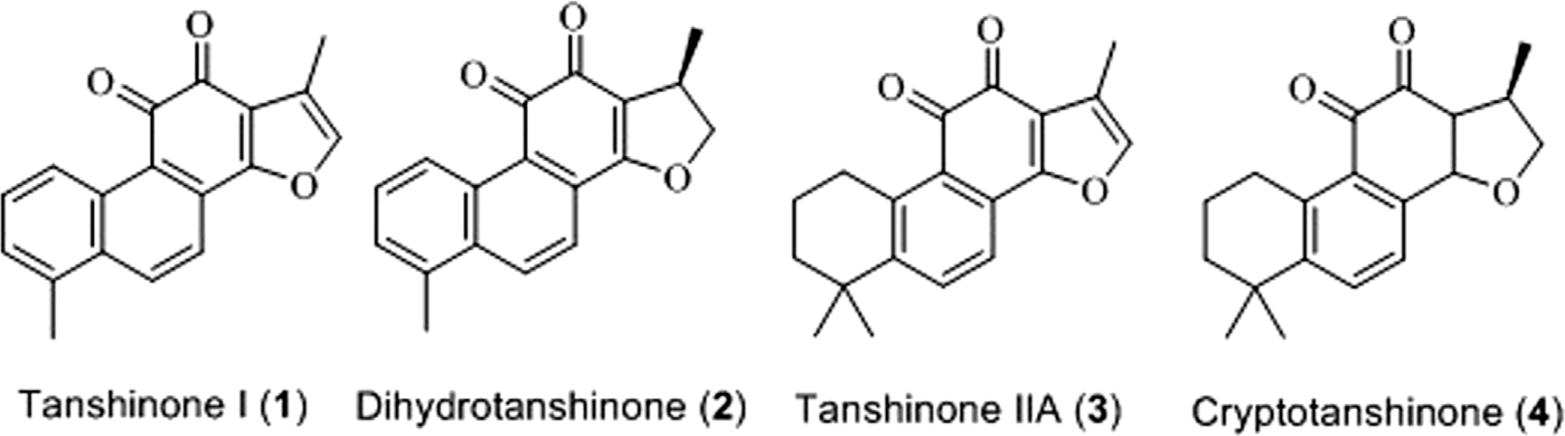
The chemical structure of tanshinone (**1–4**).

Tanshinone is a natural terpenoid compound and the main active ingredient isolated from Danshen. Modern pharmacological studies have revealed that tanshinone exhibits antiangiogenic, antioxidant, antibacterial, anti-inflammatory, and anti-tumor activities (Wang et al., 2016; Gao et al., 2017; Zhou et al., 2017). Depending on its chemical structure, tanshinone can be divided into different types, among which tanshinone I (**1**), dihydrotanshinone (**2**), tanshinone IIA (**3**), and cryptotanshinone (**4**) are considered the most important components (Hao et al., 2016; Shi et al., 2016). The chemical structures of these components are shown in Figure 1.

**FIGURE 2:**
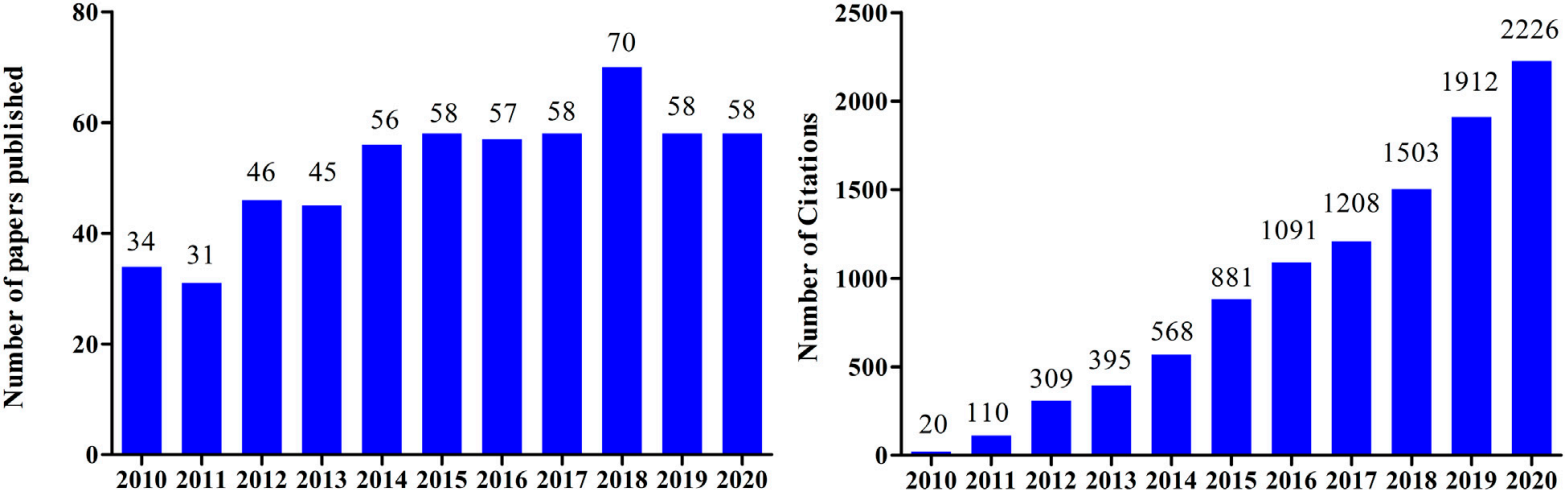
**(A)** Number of papers published between 2010 and 2020 containing the keyword “tanshinone I”, searched according to Web of Science. **(B)** Citations between 2010 and 2020 using the keyword “tanshinone I”, searched according to Web of Science.

Tanshinone I is a red crystalline powder that accounts for approximately 1.79% of the alcohol extract of *S. miltiorrhiza* roots (Lee et al., 2008). It is one of the main active components of Danshen and is widely used in the treatment of cardiovascular and cerebrovascular diseases (Liu et al., 2021). Tanshinone I also exerts antioxidant, anti-inflammatory, anti-tumor, and other pharmacological effects (Wang W. et al., 2019; Wang X. et al., 2019; Zhou et al., 2020). Tanshinone I has garnered increasing attention in the last decade, owing to its promising therapeutic effects (Figure 2).

**FIGURE 3:**
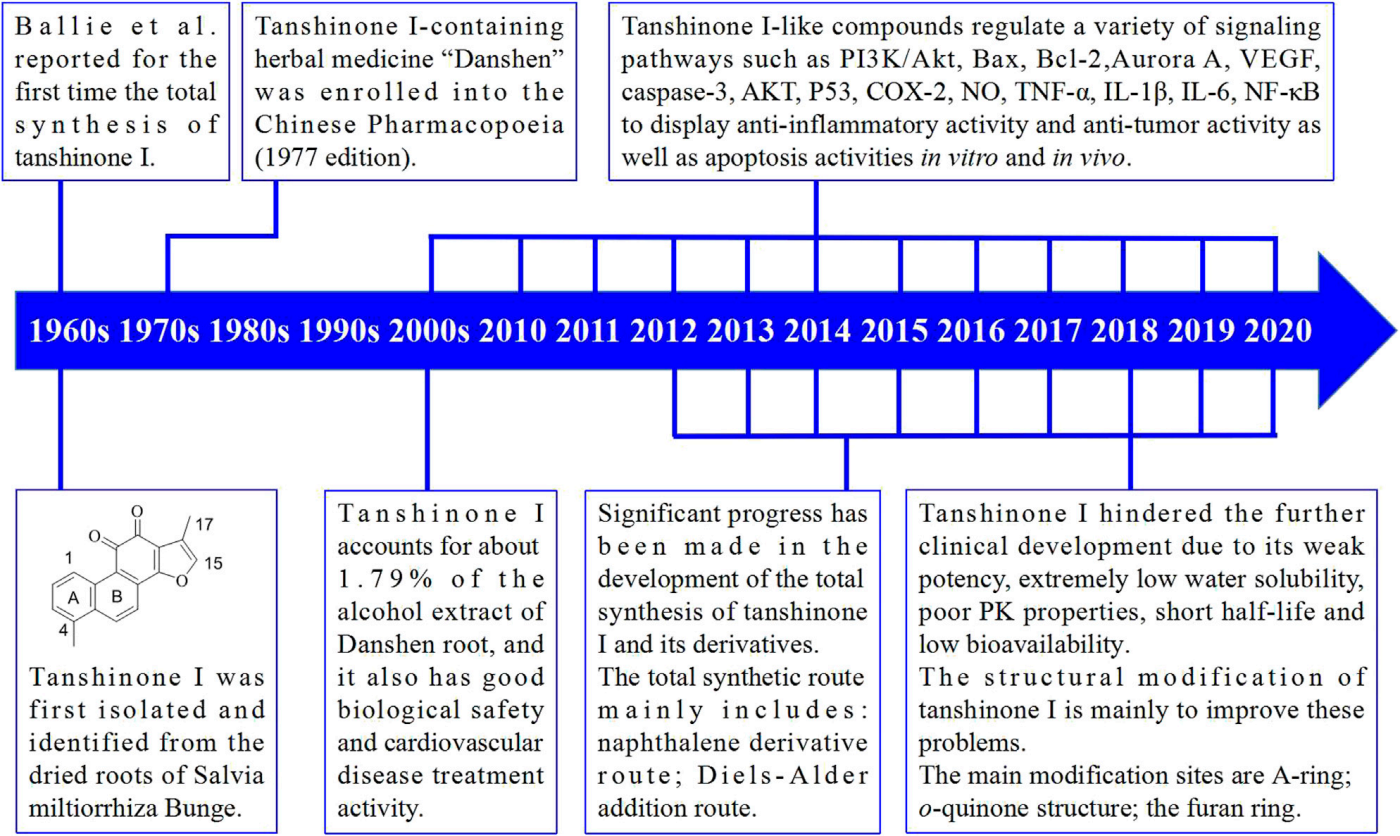
Major milestones achieved in tanshinone I-inspired drug discovery and development.

Detailed studies on the total synthesis and structural modifications of tanshinone I have been conducted. Furthermore, many synthetic routes have been developed, and many tanshinone I derivatives have been designed and synthesized. In the past few decades, substantial progress has been made in the understanding of the molecular mechanisms of action of tanshinone I and its derivatives. The main milestones achieved in the discovery and development of tanshinone I are shown in Figure 3.

In this review, we briefly summarize the biological and pharmacological data on tanshinone I and highlight the therapeutic potential and value of tanshinone I template as a promising drug development platform. We hope to provide valuable information for future pharmacological research on tanshinone I.

### 1.1 Anti-Tumor Activity

Studies have shown that tanshinone I exerts inhibitory effects on various tumors, such as lung cancer (Lee et al., 2008; Tung et al., 2013), leukemia (Liu XD. et al., 2010), colon cancer (Su et al., 2008), breast cancer (Wang Y. et al., 2015), liver cancer (Li et al., 2013), gastric cancer (Jing et al., 2016), neuroma (de Oliveira et al., 2017a), and bladder cancer (Shin et al., 2014). Tanshinone I also exerts an inhibitory effect on multidrug-resistant tumor cells (i.e., K562/A02, KB/VCR, and MCF-7/ADR) (Xu et al., 2013).

#### 1.1.1 By Inhibiting Division and Proliferation of Tumor Cells

According to Mosaddik. (2003), 25 μg/ml tanshinone I exerted a significant cytotoxic effect on lymphocytic leukemia cells (P338) and also significantly inhibited their growth and proliferation.

#### 1.1.2 By Inducing Tumor Cell Differentiation and Apoptosis

Su et al. (2008) revealed that tanshinone I can block the G0/G1 phase in human colon cancer cells (Colo 205) and cause them to undergo apoptosis mediated by caspase-3 and p53. Tanshinone I also inhibits the PI3k/Akt signalling pathway and activates telomerase activity and caspase-3 in myeloid leukemia cells to induce apoptosis (Liu JJ. et al., 2010; Liu XD. et al., 2010). Li et al. confirmed that tanshinone I can inhibit growth and promote apoptosis in leukemia and lymphoma cells. It exhibited greater anti-tumor activity than did tanshinone IIA Li et al. (2012). In the human breast cancer cell lines MCF-7 and MDA-MB-231, tanshinone I can significantly reduce the Bcl-2/Bax protein ratio to induce apoptosis (Nizamutdinova et al., 2008a). Wang and Nizamutdinova et al. revealed that tanshinone I affects the PI3K/Akt/mTOR signalling pathway through Caspase-3 activation, downregulates the level of the apoptosis inhibitor protein Bcl-2, and upregulates the level of the pro-apoptotic protein Bax to promote apoptosis in MCF-7 and MDA-MB-453 cells Nizamutdinova et al. (2008b); Wang L. et al. (2015). Ma et al. found that tanshinone I upregulates the expression of miR-32 and other related miRNAs to inhibit the AURKA gene, thereby promoting the apoptosis of non-small cell lung cancer H1299, A549, SPCA-1, HCC827, and BEAS-2B cells Ma et al. (2015).

#### 1.1.3 Blocking the Tumor Cell Cycle

Wang L. et al. (2015) found that tanshinone I can arrest MCF-7 and MDA-MB-453 cells in the S phase and cause a decrease in cyclin B levels and an increase in cyclin E and cyclin A levels. Li et al. (2013) found that tanshinone I can block non-small cell lung cancer cell lines (H1299, H23, and A549) in the S phase and inhibit DNA synthesis after cell processing.

#### 1.1.4 *In vivo* Experiments

Li et al. (2013) found that tanshinone I could inhibit proliferation in 54% of non-small cell lung cancer H1299 cells and increase apoptosis by 193% in lung cancer cells in mice. Tanshinone I was also found to inhibit lung tumor angiogenesis by 72%, reduce the weight of solid tumors by 34%; it also exhibited a dose-dependent effect. Moreover, tanshinone I did not cause changes in the food intake or body temperature of the mice during the administration period.

Lee et al. (2008) treated immunodeficient mice inoculated with lung cancer cells with tanshinone I and found that the tumor size was reduced by 85% relative to that in the control group. Tanshinone I has also been found to inhibit the formation of tumor blood vessels.

#### 1.1.5 Anti-Angiogenesis Effect

In DU145 prostate tumor mice, tanshinone I significantly inhibited tumor growth through its anti-angiogenic effect and downregulation of Aurora A (Gong et al., 2011). In this context, Wang Y. et al. (2015) found that tanshinone I can not only directly inhibit angiogenesis in tumor cells such as MCF-7, but also indirectly inhibit the angiogenesis of tumor cells by inhibiting VEGF.

#### 1.1.6 Summary of Tanshinone I Tumor Activity

Tanshinone I exhibited unique anti-tumor activity. Several studies have shown that tanshinone I had inhibitory effects on a variety of tumors *in vitro* and *in vivo*. The anti-tumor mechanisms of tanshinone include the induction of apoptosis and autophagy, regulation of the cell cycle, and inhibition of proliferation, invasion, metastasis, and angiogenesis in tumor cells. In summary, tanshinone I may be a potential anti-tumor drug and may serve as a candidate for cancer treatment. Table 1 summarizes several *in vitro* and *in vivo* studies on tanshinone I.

**TABLE 1:** Summary of *in vitro* and *in vivo* studies with tanshinone I against various cancer.

Cancer	*In vitro* studies	*In vivo* studies	Potential molecular mechanisms	Reference
Lung cancer	LD_50_ (CL1-5 cells) = 30 μg/ml	0.3 mg/kg/, tumor growth↓, angiogenesis↓, metastasis↓	Angiogenic factor IL-8↓	Lee et al. (2008)
Lung Cancer	IC_50_ (H1299, H23 and A549 cells) = 3–8 μM	200 mg/kg, tumor weight↓	Aurora A↓, angiogenesis↓, inducing apoptosis, Bcl-2↓, survivin↓, Bax↑	Li et al. (2013)
Breast cancer	MDA-MB-231 cells^a^	10 and 50 mg/kg, tumor mass volume↓, tumor metastasis↓	TNF-α↓, VEGF↓, endothelial tube formation↓	Nizamutdinova et al. (2008a)
Breast cancer	MCF-7 cells and MDA-MB-231 cells^a^	-b	Activate caspase-3, Bcl-2↓, Bax↑	Nizamutdinova et al. (2008b)
Gastric cancer	BGC823 and SGC7901 cells^a^	-^b^	Bcl-2↓, LC3-I into LC3-II↑, induce both apoptosis and autophagy, Beclin-1/VPS34↑	Jing et al. (2016)
Hematological malignancy	IC_50_ (K562 cells) = 13.52 μM (24 h), 4.70 μM (48 h), 1.59 μM (72 h), IC_50_ (Raji cells) = 4.37 μM (24 h), 1.71 μM (48 h), 1.38 μM (72 h)	-^b^	Induction of apoptosis, cell shrinkage, membrane blebbing, and karyorrhexis	Li et al. (2012)
Colorectal cancer	IC_50_ (CCD cells) = 93.6 μM (72 h), IC_50_ (SW480 cells) = 18.6 μM (72 h), IC_50_ (HCT116 cells) = 2.16 μM (72 h)	-^b^	P53↓, Aurora A↓	Lu et al. (2016)
Colorectal cancer	HCT116 and SW480 cells^a^	-^b^	cyclin D1↓, ERK1/2↓, induces ERK1/2 phosphorylation	Kim et al. (2015)
Human hepatocellular carcinoma	HepG2 and Huh7 cells^a^	-^b^	Induce G0/G1 phase arrest, trigger apoptosis, cyclin D1↓, p21↑, p53/DRAM↓, autophagy	Liu and Liu, (2020)
Cervical cancer	HeLa and C4-1 cells^a^	-^b^	induction of apoptosis, KRAS mRNA and protein↓, PI3K/AKT↓, p-AKT↓, t-AKT↑	Dun and Gao, (2019)
Colon cancer	HCT116 and HT29 cells^a^	-^b^	Bid↓, activation of p38 MAPK and caspase-3	Kim et al. (2018)

a Tanshinone I inhibited the growth of tumor cells, but did not give IC_50_.

### 1.2 Anti-Leukemia Activity

Liu XD. et al. (2010) found that tanshinone I inhibited the growth of U937, THP-1, and SHI1 leukemia cells. Furthermore, its apoptosis-inducing effect was time- and dose-dependent. Notably, the mechanism of action of tanshinone I is related to the activation of caspase-3, inhibition of hTERT mRNA expression and telomerase activity, and downregulation of survivin expression.

### 1.3 Anti-Inflammatory Activity

Kang et al. (2000) found that tanshinone I can inhibit the production of IL-12 in macrophages and IFN-γ in lymph node cells. Kim et al. (2002) revealed that tanshinone I can inhibit the secretion of prostaglandin E2 (PGE2) in macrophages but does not affect the activity or expression of COX-2. According to Tao et al. (2013a; 2013b), tanshinone I inhibits lung inflammation in mice and protects the endothelium of the trachea. Wang S. et al. (2015) demonstrated that tanshinone I can inhibit the expression or release of NO, TNF-α, IL-1β, IL-6, NF-κB, and other factors.

### 1.4 Antioxidant Activity

Based on the results of *in vivo* experiments, tanshinone I can induce the Nrf2 signalling pathway and activate the antioxidant response, thereby protecting tissues (Tao et al., 2013b). Tanshinone I prevents the inhibitory effect of hydrogen peroxide (H_2_O_2_) on TCA and mitochondrial complexes. Furthermore, in SH-SY5Y cells, tanshinone I can upregulate glutathione expression in the mitochondria and provide mitochondrial protection against H_2_O_2_ by activating Nrf2 (de Oliveira et al., 2017b).

### 1.5 Neuroprotective Effects of Tanshinone I

Kim et al. (2007); Kim et al. (2009) found that tanshinone I activates the ERK signalling pathway by increasing the levels of pCREB and pERK proteins in the hippocampus, improving learning and reducing memory impairment in mice. Lee et al. (2013) reported that tanshinone I can reduce the infarct area caused by cerebral ischemia and hypoxia in mice and reduce the death of ipsilateral brain neurons. According to Park J. H. et al. (2014) tanshinone I reduces neuronal death in the hippocampus through its anti-inflammatory effects and can increase or maintain the immune response activity and the levels of the anti-inflammatory factors IL-4 and IL-13. Zhou et al. (2011) confirmed that tanshinone I can inhibit the activity of acetylcholinesterase, indicating that tanshinone I can be used as a potential adjuvant to treat Alzheimer’s disease.

### 1.6 Other Effects of Tanshinone I

Tanshinone I also has anti-osteoporosis (Lee et al., 2005; Kim et al., 2008), and antifungal activities (Wang et al., 2007). It can improve insulin resistance in patients with type 2 diabetes (Wei et al., 2017) and inhibit allergic skin reactions (Trinh et al., 2010). Tanshinone I also protects vertebral cells from neuronal ischemia-reperfusion injury (Park JH. et al., 2014).

## 2 Total Synthesis of Tanshinone I

Total synthesis of tanshinones and their analogs has been attempted since the early 1960s (Baillie and Thomson, 1968), and many strategies have been developed. This section focuses on the main strategies employed.

### 2.1 First Total Synthesis of Tanshinone I and Diels-Alder Addition Route

**FIGURE 4:**
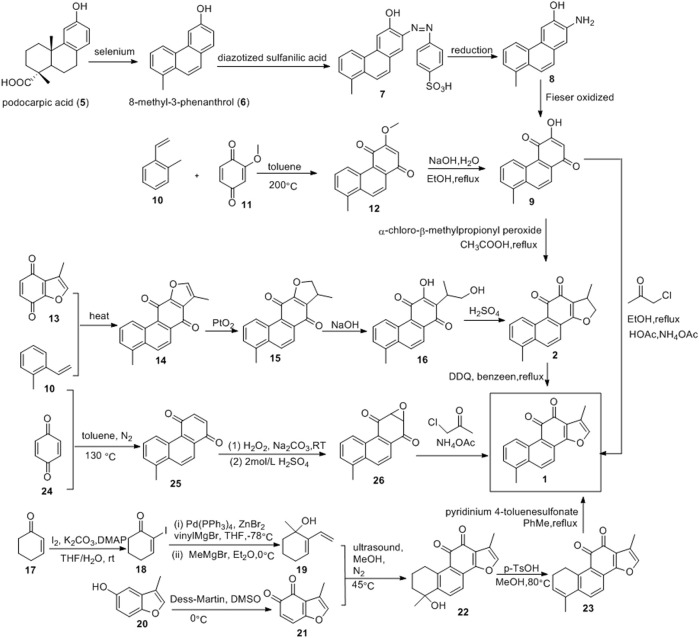
Podocarpic acid synthetic tanshinone I and Diels-Alder addition route.

The first total synthesis of tanshinone I was completed in 1968 by Baillie and Thomson, using podocarpic acid (**5**) as the starting material (Figure 4) (Baillie and Thomson, 1968). The dehydrogenation of podocarpic acid (**5**) with selenium powder resulted in 8-methyl-3-phenanthrol (**6**), which was coupled with diazotized sulfanilic acid (**7**), reduced to aminophenol (**8**), and oxidized to quinone (**9)** *via* the Fieser method. Intermediate **9** was cyclized with β-chloro-α-methylpropionyl peroxide, resulting in dihydrotanshinone, which was dehydrogenated to obtain tanshinone I, with a total yield of 34%.

Wu et al. (2017) used 2-methylstyrene (**10**) and 2-methoxy-1,4-benzoquinone (**11**) as raw materials to synthesize tanshinone I (Figure 4). Compounds **10** and **11** underwent the Diels-Alder reaction in toluene to yield compound **12**. Subsequent demethylation of compound **12** in EtOH using NaOH solution resulted in compound **9**. The Feist-Bénary reaction between compound **9** and chloroacetone in CH_3_COOH-CH_3_COONH_4_ yielded tanshinone I, with a total yield of 18.5%.

Inouye and Kakisawa also used the Diels-Alder reaction to synthesize tanshinone I (Figure 4) (Inouye and Kakisawa, 1969). *o*-Methylstyrene (**10**) and 3-methylbenzofuran-4,7-quinone (**13**) were heated without solvent to obtain phenanthrenequinone (**14)**. Compound **14** was then precipitated with platinum oxide in acetic acid to obtain compound **15**, which was then hydrolyzed with potassium hydroxide to obtain compound **16**. Compound **16** was acidified with concentrated sulfuric acid to obtain dihydrotanshinone. Finally, DDQ was used to process dihydrotanshinone to obtain tanshinone I with a total yield of approximately 4%.

Wang et al. (2018) synthesized tanshinone I using a simple and efficient method (Figure 4). 2-cyclohexenone (**17**) was subjected to α-iodination under K_2_CO_3_/DMAP/I_2_ conditions to obtain iodoketene **(18)**. Compound **20** was cross-coupled, catalyzed by palladium, and then subjected to Grignard addition with methylmagnesium bromide to obtain hydroxydiene (**19)**. Benzofuran-4,5-dione dienophile (**21**) was prepared *via* the oxidation of 3-methyl-benzofuran-5-ol (**20**). Compounds **19** and **21** underwent the Diels-Alder reaction to obtain compound **22**. Compound **22** underwent an elimination reaction catalyzed by p-toluenesulfonic acid to obtain the key intermediate **23**. The aromatization of intermediate **23** yielded tanshinone I.

Yang et al. (2016) reported a three-step synthesis route for tanshinone I, which includes the Diels-Alder reaction, ∆^2^-Weitz-Scheffer-type epoxidation, and the Feist–Bénary reaction (Figure 4). First, the Diels–Alder reaction between *o*-methylstyrene (**10**) and *p*-benzoquinone (**24**) directly yielded 1,4-phenanthrenedione (**25**). Thereafter, compound (**25**) was oxidized with hydrogen peroxide to obtain the epoxy group (**26**). Finally, the dicarbonyl compound was condensed with α-haloketone, and the furan ring was formed *via* elimination to obtain tanshinone I. This method can be used to obtain tanshinone I and its derivatives with different substituents.

### 2.2 Routes of Benzene or Naphthalene Derivatives

**FIGURE 5:**
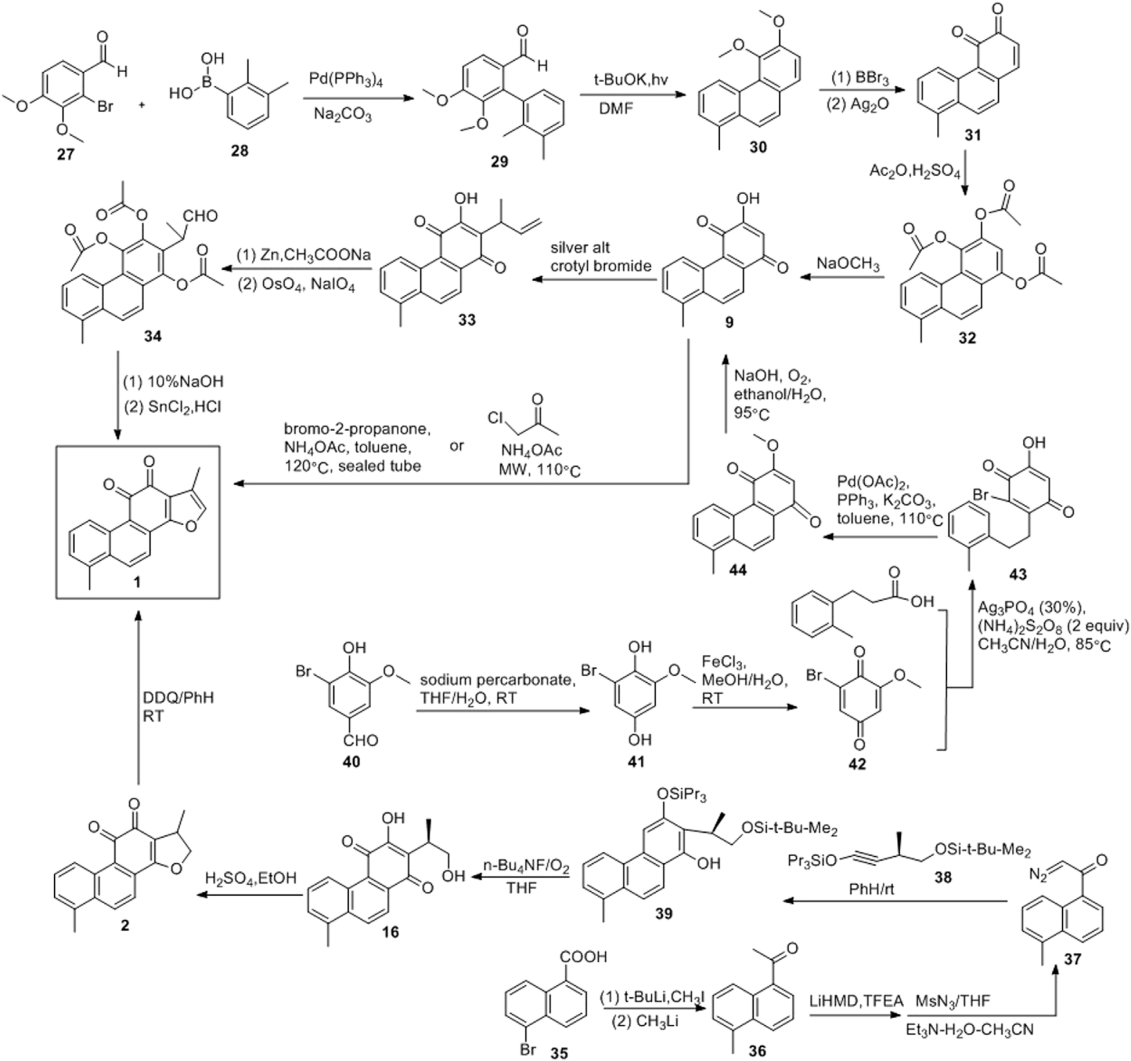
Benzene or naphthalene derivatives addition route.

De Koning et al. (1998) developed a photochemical cyclisation method that can be used to produce key intermediates (**30**), as shown in Figure 5. The coupling reactions of compounds **25** and **26** yielded **27**. Treatment of **27** with potassium tert-butoxide under simultaneous irradiation with a high-pressure mercury lamp yielded 56% of the intermediate. In 1974, Hout and Brassard (1974) used **20** to obtain tanshinone I. Demethylation and oxidation of compound **30** resulted in quinone **31**, and its acetylation led to **32**. Compound **9** was obtained *via* reaction with sodium methoxide, followed by the addition of methallyl to produce **33**. Acetylation and oxidative cleavage of compound **33** resulted in the formation of the aldehyde **34**. After alkaline hydrolysis of compound **34**, the acid promoted cyclization, resulting in tanshinone I.

Rubottom and Kim (1983) developed a photochemical aromatic cyclization strategy for the synthesis of tanshinone I (Figure 5). 5-Bromo-1-naphthoic acid (**35**) was first converted to methyl ketone **36**. Thereafter, the diazonium transfer method was employed to synthesize key diazoketone **37** using **36**. Compounds **37** and **38** produced phenol 34 under irradiation with a low-pressure mercury lamp (254 nm). The treatment of **39** with tetra-n-butylammonium fluoride in the presence of oxygen resulted in **16**, which was converted into dihydrotanshinone with concentrated sulfuric acid. The dehydrogenation of dihydrotanshinone with DDQ yielded the final product, tanshinone I. The synthetic route was divided into six steps, and tanshinone I was obtained in a 33% yield.

Researchers (Jiao et al., 2014; Foulkes et al., 2020) synthesized tanshinone I using a relatively mild method (Figure 5). Sodium percarbonate was used to oxidize 5-bromovanillin (**40**), and the ester functional group was hydrolyzed to obtain 1,4-hydroquinone (**41**). The subsequent oxidation of **41** with FeCl_3_ yielded **42**. Using 0.3 equivalents of AgNO_3_ and 2 equivalents of (NH_4_)_2_S_2_O_8_, the decarboxylation radical alkylation reaction of 3-phenylpropionic acid with **42** proceeded smoothly to obtain compound **43**. Compound **43** was reacted with Pd (OAc)_2_ (0.45 equivalent), PPh_3_ (0.8 equivalent), and K_2_CO_3_ (3 equivalent) under reflux with toluene for 12 h. Owing to intramolecular cyclisation, compound **44** were obtained. Compound **44** was treated with an aqueous sodium hydroxide solution under reflux with EtOH under O_2_ atmospheric pressure to obtain the key intermediate **9**. Finally, anhydrous toluene was used as the solvent, and compound **9** was treated with bromo-2-acetone and NH_4_OAc at 120°C in a sealed tube to obtain tanshinone I (yield: 35%).

### 2.3 Summary of the Total Synthesis of Tanshinone I

Since the report of the first total synthetic route of tanshinone I in 1968, several methods for the total synthesis of tanshinone I have been consecutively developed. The most important route is the Diels-Alder addition route. Intermediate **9** is the most important intermediate in this route, and the ring is closed with bromo-2-acetone to form a furan ring, resulting in tanshinone I. In addition, tanshinone I can be obtained by the oxidation of dihydrotanshinone.

## 3 Structural Modification of Tanshinone I

Tanshinones, including tanshinone IIA, tanshinone I, and cryptotanshinone, represent a large class of tetracyclic furanquinone diterpenoids isolated from the traditional Chinese medicine Danshen, which exhibits a variety of pharmacological activities (Wang et al., 2007; Dong et al., 2011).

Further clinical development of tanshinone I was hindered by its weak potency, extremely low water solubility (Yu et al., 2012), poor PK properties (Park et al., 2008; Liu Y. et al., 2010), short half-life, and low bioavailability (Wu and Yang, 2014). Therefore, it was necessary to modify the structure of tanshinone I to improve its biological activity and water solubility.

### 3.1 Derivatization Site of Tanshinone I

In recent years, studies on tanshinone I have led to improved water solubility through structural modifications, thereby improving its bioavailability. The structure of tanshinone I provides several sites for derivatization. The modified sites of tanshinone I include *o*-quinone, C-17, and C-15 of the furan ring.

#### 3.1.1 Structural Modification of *o*-Quinone Sites

**FIGURE 6:**
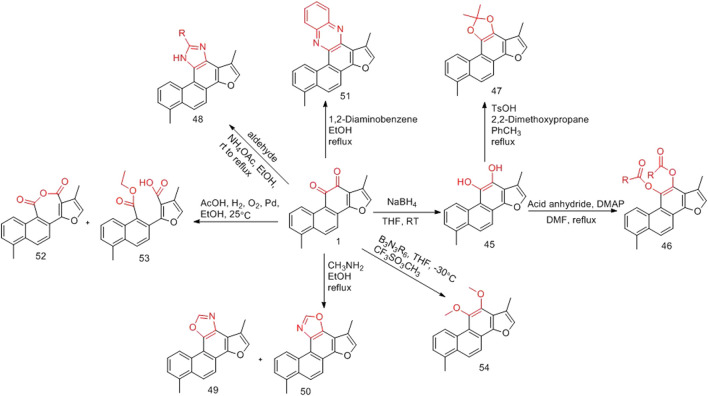
Structural modification of *o*-quinone sites.

The first modification method of the o-quinone structure of tanshinone I involves the reduction of *o*-quinone (**45**) (Foulkes et al., 2020), followed by acylation of the phenolic hydroxyl group (**46**) (Foulkes et al., 2020) or alkylate (**47**) (Foulkes et al., 2020). The second modification method uses amine compounds to react with 11 and 12 o-dicarbonyl groups to generate imidazole (**48**) (Li et al., 2018), oxazole (**49**, **50**) (Li et al., 2018), and pyrazine (**51**) (Li et al., 2018). The third modification method involves the oxidation of the *o*-quinone structure to form acid anhydride (**52**) (Zhang et al., 2013); however, this method results in a by-product of ring opening (**53**) (Li et al., 2014). Finally, the *o*-quinone structure can be directly transformed into an *o*-methoxy structure (**54**) (Li et al., 2020) (Figure 6).

#### 3.1.2 Structural Modification of Furan Ring, A Ring and B Ring of Tanshinone I

**FIGURE 7:**
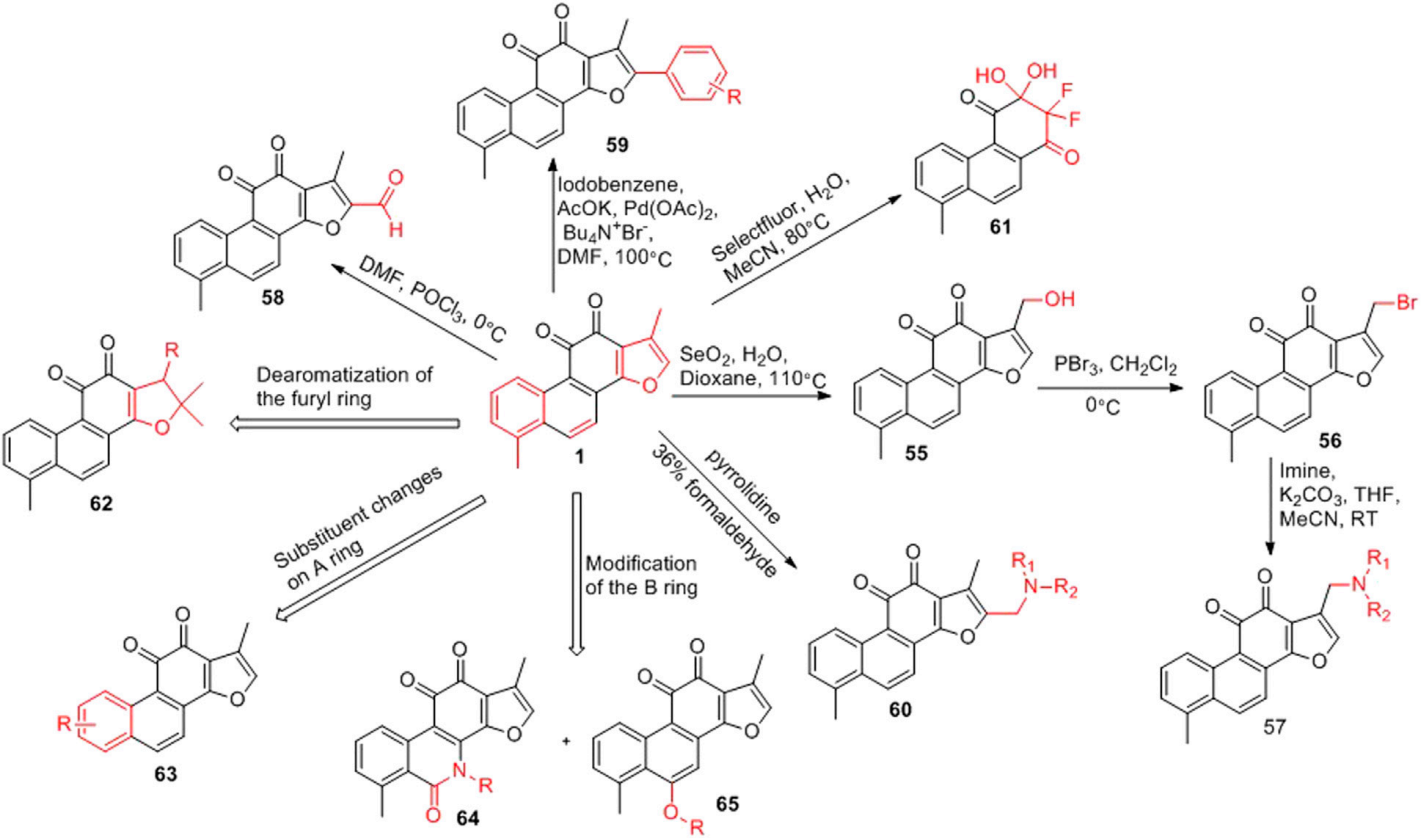
Structural modification of furan ring, A ring and B ring of tanshinone I.

The furan ring of tanshinone I is a hot spot for modification (Figure 7). The C-17 position of the furan ring is oxidized to introduce a hydroxyl group (**55**) (Ding et al., 2018). The hydroxyl group can be brominated (**56**) (Ding et al., 2018), and bromine can be substituted with various imines to obtain new derivatives (**57**) (Ding et al., 2018). An aldehyde group (**58**) can also be introduced at the C-15 position *via* the Vilsmeier–Haack reaction (Wang et al., 2017b); while a phenyl group (**59**) can be introduced at the C-15 position *via* a coupling reaction (Jiao et al., 2015). Furthermore, an aminomethylated product (**60**) can be obtained *via* the Mannich reaction (Wang D. et al., 2015). Tanshinone I can be used to obtain a unique α,α-difluoro β,β-dihydroxy-p-quinone (**61**) through a fluorination reaction (Li et al., 2017).

In the total synthesis of tanshinone I, changing the reaction conditions can result in derivatives with great structural changes, including dearomatization of the furan ring of tanshinone I (**62**) (Ding et al., 2018), changes in the substituents on the phenyl group of the A ring (**63**) (Jiao et al., 2014; Foulkes et al., 2020), and in the structure of the B ring (**64**, **65**) (Ding et al., 2018).

### 3.2 Synthetic Route of 2-Aryl Derivative of Tanshinone I

**FIGURE 8:**
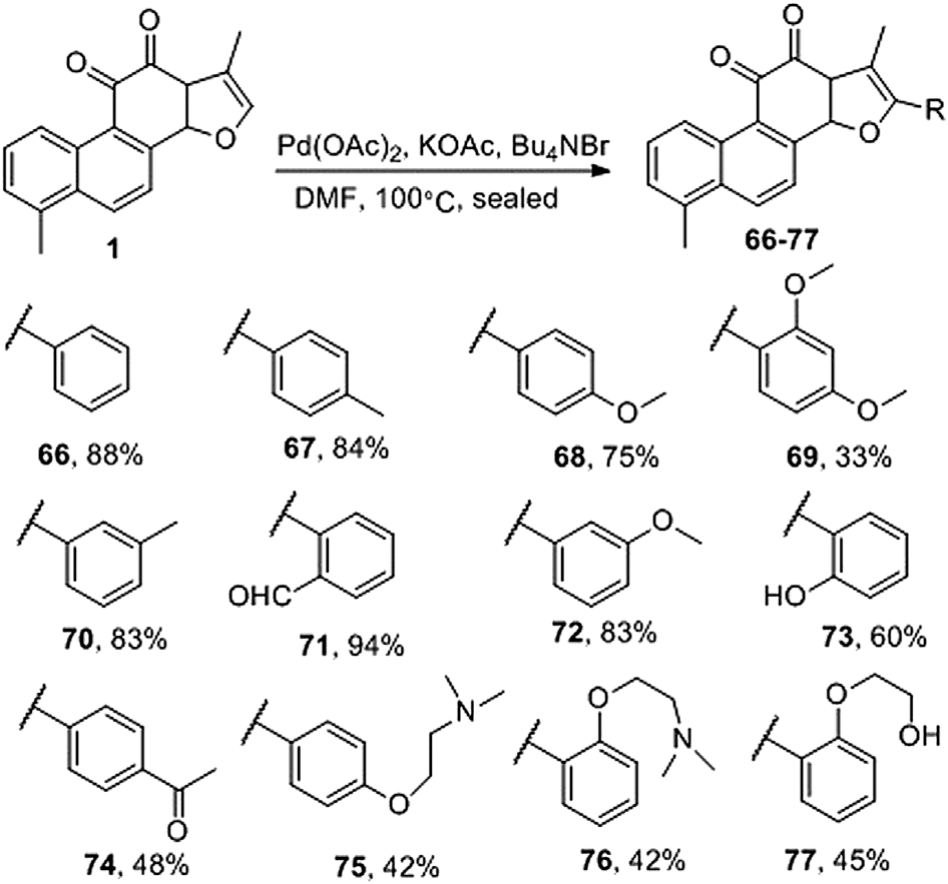
Synthetic route of 2-aryl derivative of tanshinone I (**66–77**).

Jiao et al. (2015) directly activated Csp2-H on the a-position of the furan ring of tanshinone I and produced palladium-catalyzed carbon–carbon coupling to prepare the natural product tanshinone I 2-arylated derivative (**66–77**) (Figure 8). Pharmacological activity has not yet been reported.

**FIGURE 9:**
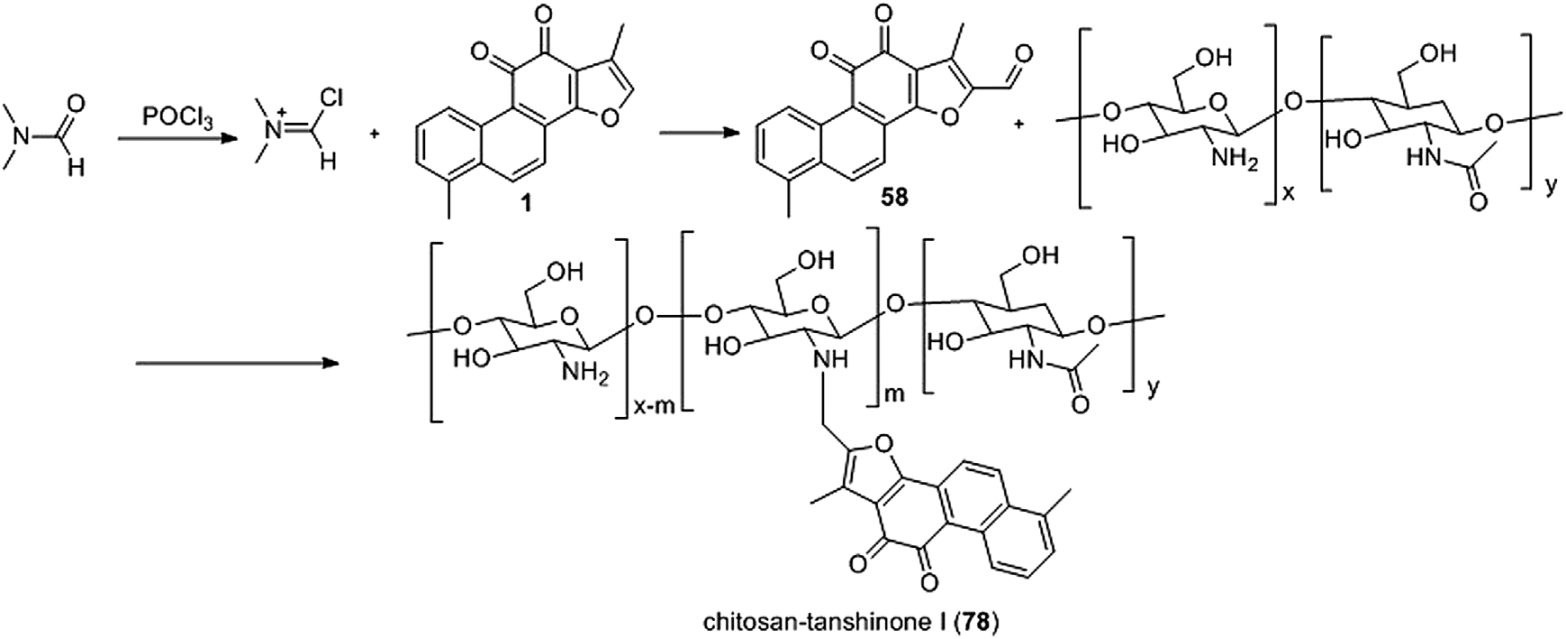
Synthetic chitosan-tanshinone I (**78**).

Wang et al. (2017a) synthesized a novel water-soluble low-molecular chitosan (LMC) tanshinone I derivative (**78**) (Figure 9). Derivative **78** effectively reduced the A549 and 7901 cell viability rate, inhibited metastasis, induced apoptosis, and dissipated mitochondrial membrane potential (ΔΨm).

### 3.3 Synthetic Route of *o*-Quinone of Tanshinone I

**FIGURE 10:**
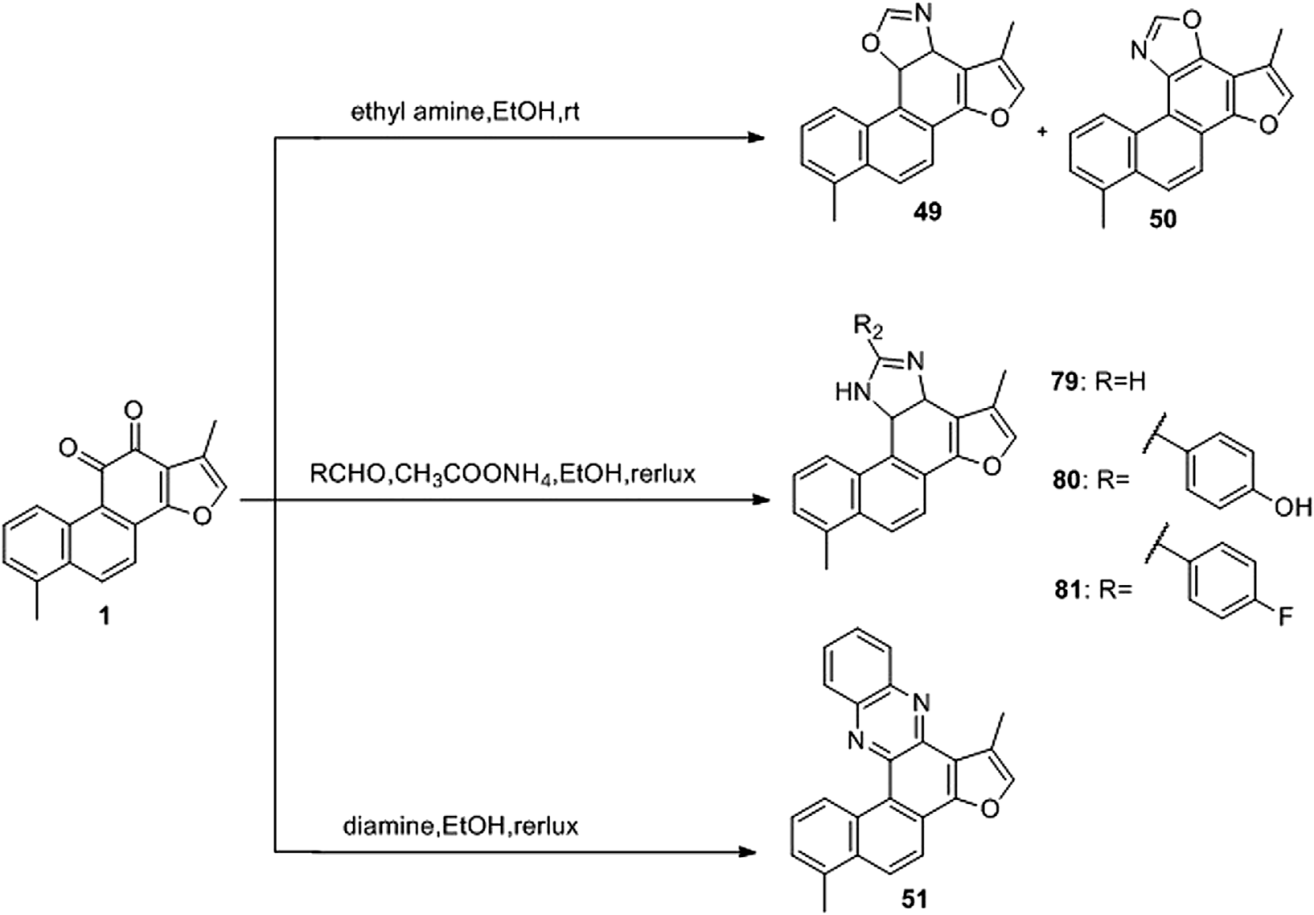
Synthetic route of *o*-quinone of tanshinone I (**49–51**, **79–81**).

Oxazoline (Pfeiffer et al., 2009), imidazole (Komodzinski et al., 2013), and pyrazine (Rajule et al., 2012) derivatives are used in oncology. Li et al. (2018) used tanshinone I as a raw material to synthesize a library of C-11/C-12 small nitrogen heterocyclic derivatives characterized by oxazole, imidazole, and pyrazine rings *via* a simple method (Figure 10). Consequently, six new derivatives were synthesized (**49–51**, **79–81**). Based on pharmacological experiments, four derivatives (**51**, **79–81**) showed moderate cytotoxic activity against five human cancer cell lines *in vitro*. Furthermore, compounds **50** and **79** exhibited activity against CNE cells. Therefore, the imidazole derivatives displayed higher activities than did the oxazoline and pyrazine alternatives. Among the imidazole derivatives, aryl substitution (**81**, **80**, **51**) on the oxazole ring has been proven to be effective. Compared to tanshinone I, the stronger biological activity of **80** indicates that this modification strategy is promising.

### 3.4 Synthetic Route of 12-Position of Tanshinone I

**FIGURE 11:**
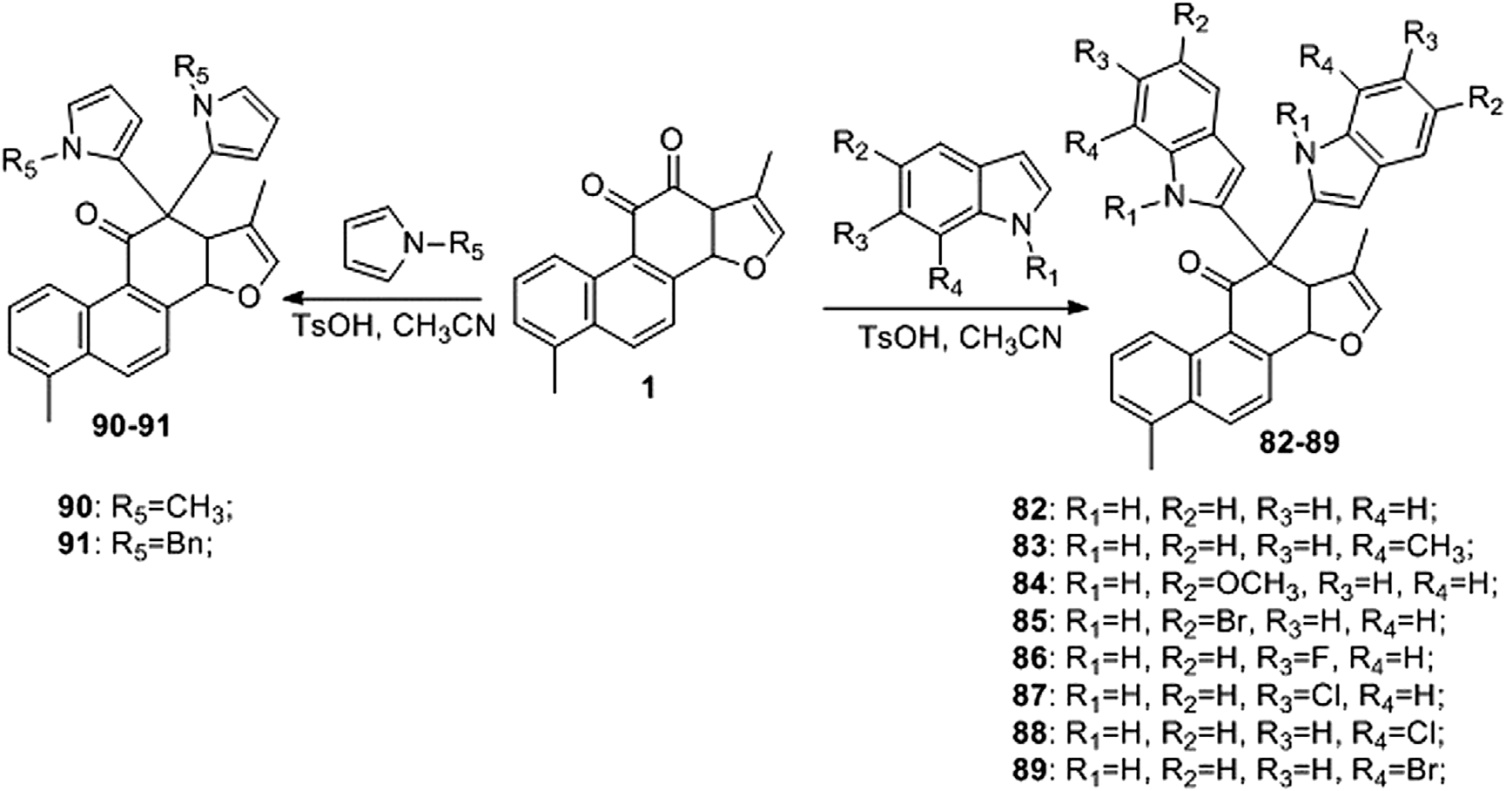
Synthetic route of 12-position of tanshinone I (**82–91**).

Liu et al. (2017) developed an efficient and straightforward methodology for the synthesis of novel tanshinone I-substituted bis (indolyl/pyrrolyl) methane scaffolds **82–91** through the TsOH-catalysis-enabled addition of indoles or pyrroles with tanshinone I, based on a molecular hybridization strategy (Figure 11). Good product yields (up to 81%) were obtained. Furthermore, their biological activities against the human leukemia cell line K562, human prostate cancer cell line PC3, and human lung cancer cell line A549 were preliminarily demonstrated using *in vitro* assays.

Compounds **50** and **79**–**88** are all derivatives of tanshinone I, and their IC_50_ values for cancer cells are summarized in Table 2.

**TABLE 2:** The anti-proliferation effect of tanshinone I derivatives on a variety of cancer cell lines (IC_50_ μM).

Compounds	IC_50_ (μM)					
	HeLa	K562	MCF-7	PC 3	CNE	A549
1	16.66	6.12	21.82	5.15	11.36	-^a^
50	19.3	41.4	14.7	>100	6.1	-
79	5.6	10.7	7.8	46.6	8.5	-
80	6.8	2.6	8.4	15.3	15.7	-
81	11.6	3.8	10.8	21.2	22.9	-
82	-	23.5	-	43.6	-	23.7
**83**	-	72.6	-	>100	-	77.6
**84**	-	34.9	-	39.8	-	69.9
**85**	-	34.9	-	39.8	-	69.9
**86**	-	45.6	-	54.2	-	45.6
**87**	-	35.2	-	76.7	-	39.1
**88**	-	>100	-	>100	-	43.6

a Not available.

Bold values represent tanshinone I-derived numbers.

Reports have been published on the structural modification of tanshinone I, including the introduction of groups at the C-15 position, such as phenyl (Jiao et al., 2015). The introduction of other substituents on the A ring (Jiao et al., 2014; Foulkes et al., 2020), or *o*-quinone moiety forms a heterocyclic ring such as an imidazole ring (Li et al., 2018). However, most of these derivatives do not exhibit strong *in vitro* anti-tumor activity. Therefore, a more extensive and systematic structural modification of tanshinone I is required, and new tanshinone I derivatives should be developed to improve the anti-tumor efficacy of tanshinone and improve its water solubility, metabolic stability, and pharmacokinetic properties.

**FIGURE 12:**
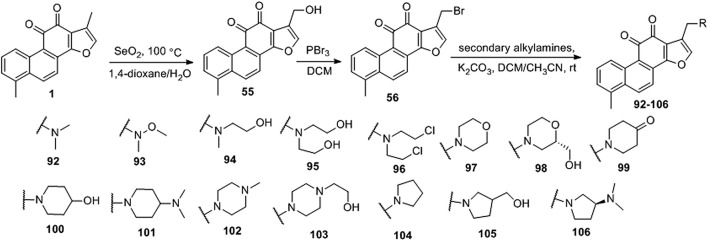
Introducing a nitrogen-containing functional group at the C-17 position.

**FIGURE 13:**
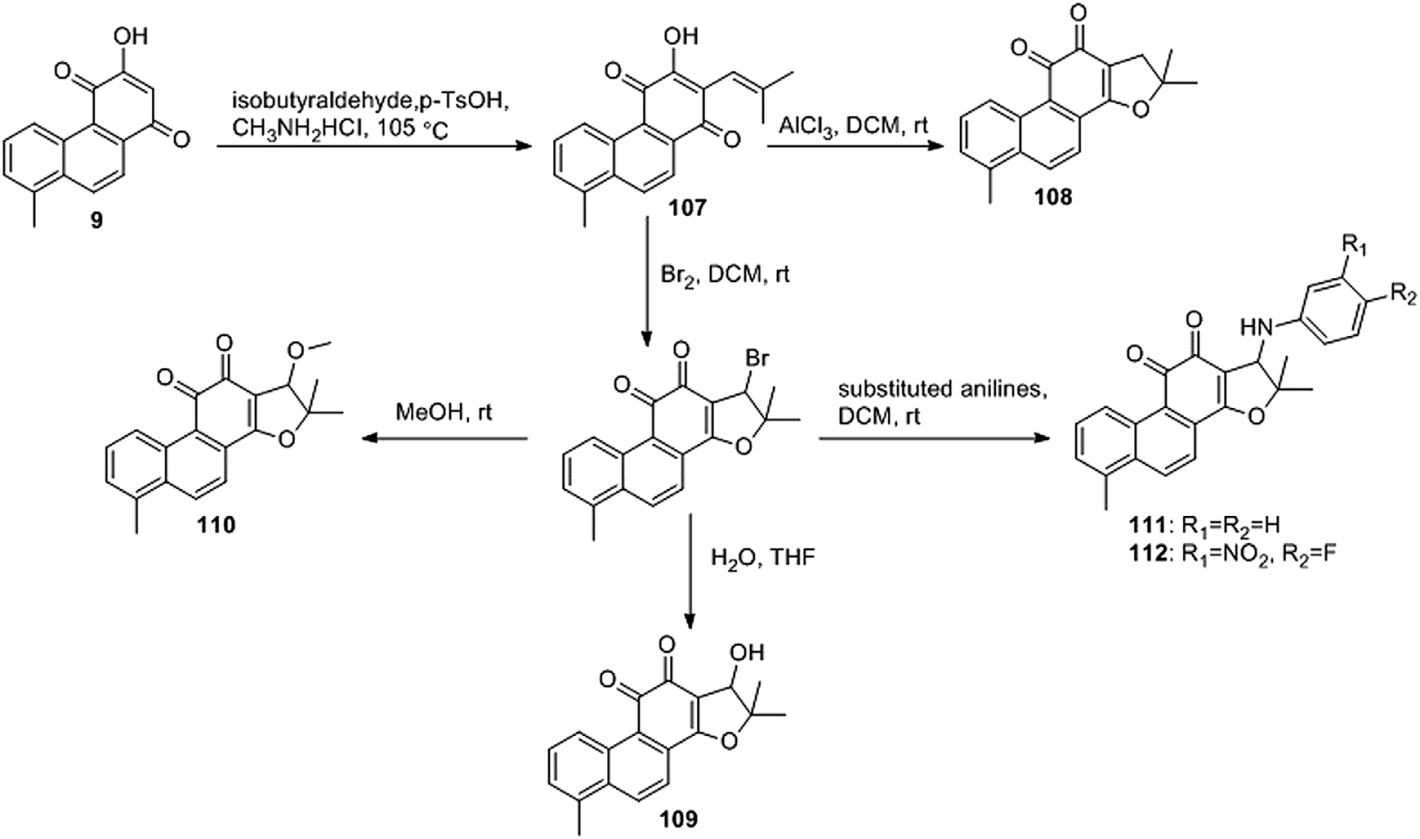
Furan ring dearomatization.

**FIGURE 14:**
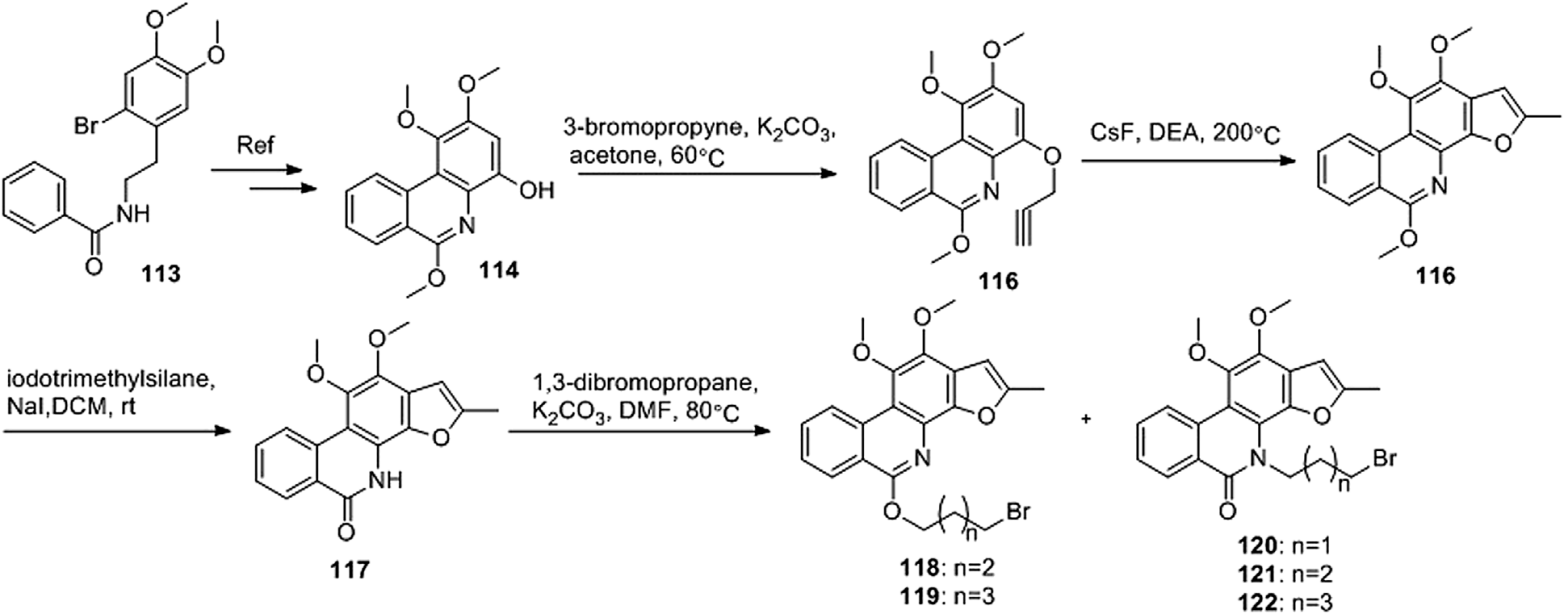
Synthesize the key intermediates **118–122**.

**FIGURE 15:**
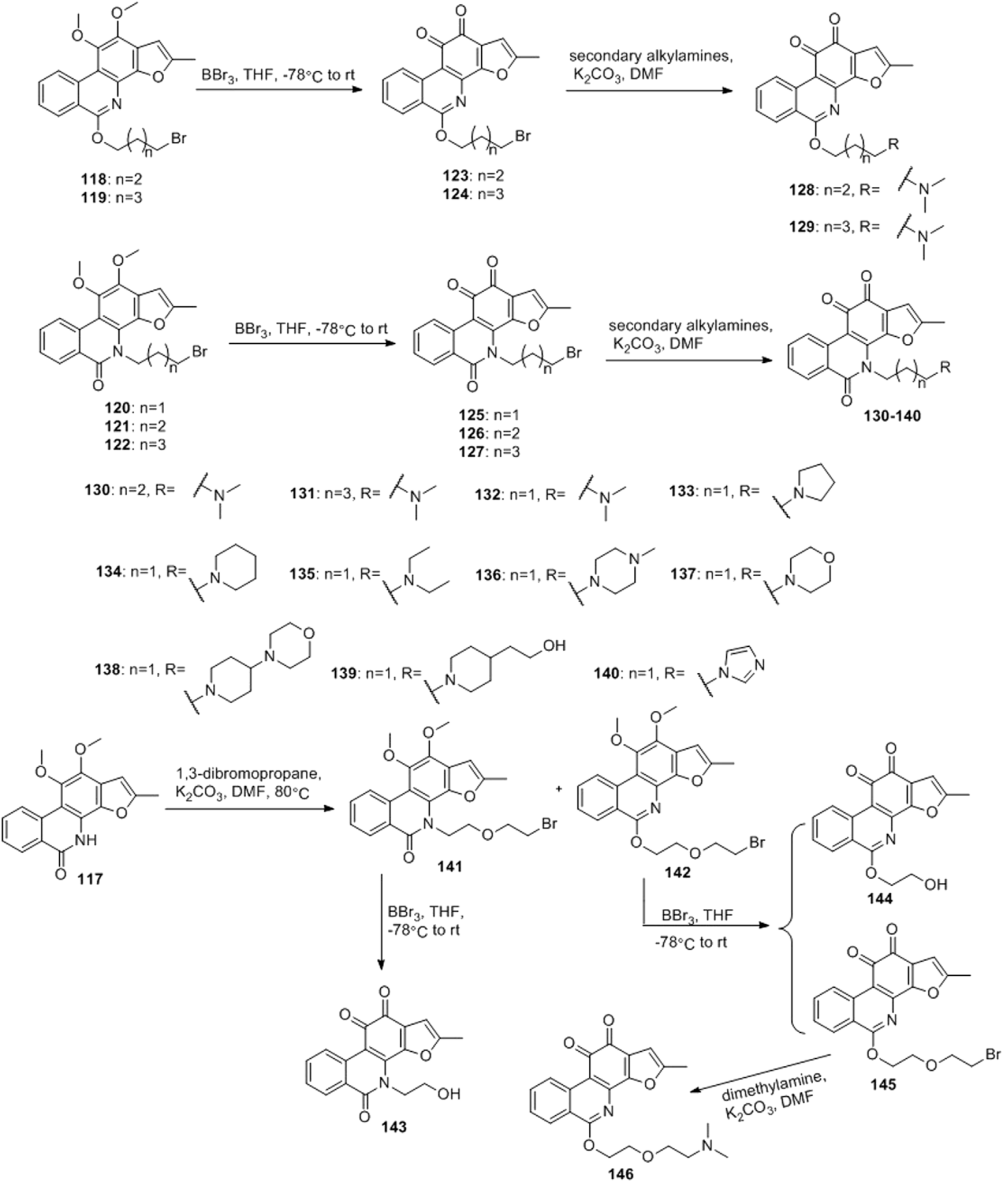
Anchor the side chain on the N- or O- moiety.

Ding et al. (2018) designed four series of tanshinone analogs through 1) the introduction of a nitrogen-containing functional group at the C-17 position to obtain derivatives **92–106** with increase water solubility and molecular flexibility (Figure 12); 2) dearomatization of the metabolically unstable furyl ring to obtain derivatives **108–112**; this method can reduce the aromaticity of tanshinone I and increase its molecular stability (Figure 13); 3) the key intermediates **118–122** (Figure 14) were synthesized, and the naphthylene A/B ring was replaced with isoquinolinone or isoquinoline. The side chain was anchored on the N- or O-moiety to obtain derivatives **128–140**, **143**, **144**, and **146** (Figure 15).

The growth-inhibitory effect of synthetic tanshinone I derivatives on squamous cell carcinoma KB cells and corresponding vincristine-resistant KB/VCR cells was assessed *via* the sulforhodamine B (SRB) assay, as described in the *in vitro* screening protocol (experimental section), and the *in vitro* anti-proliferative activity results are summarized in Table 3.

**TABLE 3:** Antiproliferative effects of tanshinone I derivatives (**55**, **92–106**, **108–112**, **128–140**, **143–144**, and **146**) on human cancer cell lines.

Compound	IC_50_ (μM)^a^		Compound	IC_50_ (μM)^a^	
	KB	KB/VCR		KB	KB/VCR
**55**	3.22 ± 0.65	2.95 ± 0.69	**143**	>20	>20
**92**	1.11 ± 0.30	0.51 ± 0.03	**144**	>20	>20
**93**	8.12 ± 3.19	5.71 ± 1.55	**128**	>20	>20
**94**	1.50 ± 0.09	1.02 ± 0.07	**129**	15.27 ± 0.10	1.99 ± 0.25
**95**	1.64 ± 0.30	1.43 ± 0.06	**130**	4.05 ± 0.05	2.47 ± 0.78
**96**	9.43 ± 6.18	10.88 ± 4.84	**131**	13.84 ± 1.37	5.17 ± 0.69
**97**	>20	>20	**132**	0.71 ± 0.16	0.92 ± 0.02
**98**	4.97 ± 3.01	2.41 ± 0.15	**133**	3.40 ± 0.78	2.64 ± 1.07
**99**	9.71 ± 4.88	4.74 ± 0.23	**134**	1.12 ± 0.49	1.53 ± 0.70
**100**	1.54 ± 0.31	1.11 ± 0.14	**135**	0.12 ± 0.02	0.33 ± 0.04
**101**	3.80 ± 0.40	3.82 ± 0.08	**136**	10.38 ± 3.43	3.52 ± 0.99
**102**	5.22 ± 0.77	13.12 ± 1.23	**137**	2.30 ± 0.54	1.56 ± 0.65
**103**	2.68 ± 0.26	4.41 ± 0.02	**138**	0.34 ± 0.22	1.72 ± 0.65
**104**	1.59 ± 0.16	1.79 ± 0.25	**139**	0.87 ± 0.11	1.49 ± 0.02
**105**	1.27 ± 0.26	0.91 ± 0.21	**140**	17.24 ± 1.72	10.07 ± 4.56
**106**	2.61 ± 0.08	1.70 ± 0.03	**146**	3.78 ± 1.23	1.65 ± 0.01
**108**	2.62 ± 0.23	2.37 ± 0.01	**1**	5.87 ± 0.70	4.40 ± 0.12
**109**	1.63 ± 0.03	1.24 ± 0.23	VCR (nM)	0.72 ± 0.16	357.51 ± 29.89
**110**	2.19 ± 0.49	2.02 ± 0.76			
**111**	>20	>20			
**112**	>20	>20			

a IC_50_ values are shown as the mean ± SEM (μM) from two independent experiments.

Bold values represent tanshinone I-derived numbers.

Compared to tanshinone I, compound **55** exhibited a more potent anti-proliferative effect on the two cancer cell lines tested. Most 17-amino compounds exhibited significant anti-proliferative activity against KB and KB/VCR cells. Among the 17-amino derivatives **92–106**, compound **92** exhibited the most potent anti-proliferative activity against the two cancer cell lines tested, with IC_50_ values of 1.11 and 0.51 μM, respectively.

Among the furfur-ring dearomatized analogs (**108–112**), compound **108** exhibited moderate inhibitory activity against the proliferation of the two cancer cells (KB and KB/VCR cells) tested, with an IC_50_ value of approximately 2.0 μM. Compound **109** was the most active, with IC_50_ values of 1.63 and 1.24 μM, respectively. Pharmacological experiments showed that the introduction of functional groups into the B ring or furanyl 17-methyl group, which increased water solubility and significantly enhanced the anti-proliferative activity in KB and KB/VCR cells. It also significantly improved water solubility and metabolic stability.

Among the series of 7-aza derivatives with different alkyl side chains (**128–149**, **143, 144** and **146**), compound **132** showed significant potency improvement against KB and KB/VCR cells with IC_50_ values of 0.71 and 0.92 μM, respectively. Extending the length of the N- or O-alkyl side chains resulted in decreased cellular efficacy, especially in KB cells. The inhibitory activity of the O- or N-aminopentyl derivatives **129** and **131** was approximately 2- to 7-fold higher in KB/VCR cells than in KB cells. In conclusion, the N-aminopropyl side chain was determined to be the optimal side chain for attachment to the 7-aza B ring. Of the derivatives **133–140** with various N-aminopropyl side chains, most exhibited potent anti-proliferative activity with low micromolar or sub-micromolar IC50 values. Among all derivatives, the derivative **135** showed the strongest anti-proliferative activity, with an IC_50_ of 0.12 and 0.33 µM for KB and KB/VCR cells, respectively. The effect on KB and KB/VCR cells was approximately 49- and 13-fold higher than that of tanshinone I. Furthermore, compound **135** significantly improved drug-like properties, enhanced water solubility (15.7 mg/ml), improved metabolic stability of liver microsomes, and improved pharmacokinetic characteristics (T_1/2_ = 2.58 h; F = 21%). Compound **135** induced caspase 3/7-dependent apoptosis in HCT116 cells in a concentration-dependent manner and mediated apoptosis. In nude-mouse experiments, compound **135** was administered at 10 mg/kg; this concentration significantly inhibited the growth of HCT116 xenografts but did not reduce the body weight of the mice.

### 3.5 Synthesis of 2-(N-Pyrrolidine-Alkyl) Tanshinone I (147)

**FIGURE 16:**
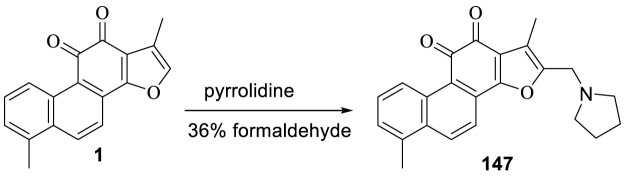
Synthesis of 2-(N-pyrrolidine-alkyl) tanshinone I (**147**).

Wang D. et al. (2015) synthesized 2-(*N*-pyrrolidine-alkyl) tanshinone I (**147**) with tanshinone I and tested its antibacterial activity (Figure 16). Tanshinone I showed no antibacterial activity against Gram-positive and Gram-negative bacteria (minimum inhibitory concentration >128 μg/ml). Compound **147** had a selective inhibitory effect on Gram-positive bacteria, with a minimum inhibitory concentration ranging from 8 to 16 μg/ml; however, this compound was non-toxic to Gram-negative bacteria (minimum inhibitory concentration >64 μg/ml).

### 3.6 Summary on Structure-Activity Relationship of Tanshinone I Analogues

**FIGURE 17:**
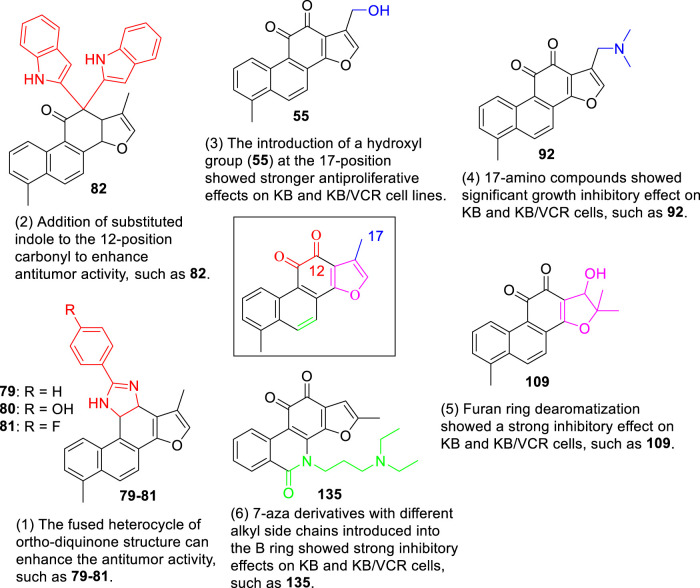
Graphical depiction of the general structural anticancer activity relationship of tanshinone I derivatives.

In summary, this chapter reviews the latest progress in structural modification, aiming to explore the biological activities of various tanshinone I analogs, mainly the anticancer activity is the most studied. The synthesis of various tanshinone I analogs and the cumulative results of their anticancer effects on various malignant cells established meaningful SARs, as shown in Figure 17. In short, modification of the C-17 position can significantly improve the anticancer efficacy and water solubility of tanshinone I. Modification of the ortho-diquinone structure and dearomatization of the furan ring can increase the structural diversity and enhance the anticancer activity. The 7-nitrogen fragments introduced into the B-ring with different alkyl side chains enhanced the anticancer activity, significantly improved the drug-like properties, enhanced water solubility, and improved the metabolic stability and pharmacokinetic characteristics of the liver. There have been relatively few studies on the modification activity of the A-ring, and no useful SAR has been established for the A-ring region. In conclusion, existing synthesis studies provide useful and efficient modification strategies for the further development of tanshinone I derivatives with enhanced biological activities and pharmaceutical properties.

## 4 Conclusion

Natural products are valuable sources of molecular diversity for drug discovery. *S. miltiorrhiza* is a Chinese medicinal plant that exerts stable pharmacological effects in the treatment of cardiovascular diseases. Based on an in-depth study of *S. miltiorrhiza*, its components were isolated, and tanshinone I was reported to be an important component.

In the past decade, the biological potential of tanshinone I in leukemia as an anti-inflammatory and anticancer agent has attracted significant interest. A large number of studies have shown that tanshinone I can achieve anti-tumor effects in a variety of ways, including inhibiting tumor cell division and proliferation, inducing tumor cell differentiation and apoptosis, and blocking the tumor cell cycle. Therefore, tanshinone I has a great anticancer potential. However, its efficiency and clinical potential are affected by its low water solubility, low bioavailability, short half-life, and poor pharmacokinetic properties. Therefore, to avoid these problems, various derivatives were designed and synthesized to identify highly effective drugs. A variety of potential targets and signalling pathways related to tanshinone I and its partial derivatives have been identified, providing new candidate compounds for more effective anticancer drugs. However, there are still some questions and new directions for future development to advance tanshinone I analogs into viable treatments:

1) Although Danshen and tanshinone I have received great attention in the past decade, their exact molecular mechanisms in the treatment of cancer and other diseases remain to be elucidated. Current research results show that the efficacy and clinical potential of tanshinone I are affected by low water solubility, low bioavailability, short half-life, and poor pharmacokinetic properties, and it is difficult to apply tanshinone I in clinical practice. We hope that subsequent studies will map the complete signalling network associated with tanshinone I to facilitate future research on new drugs for potential clinical indications.
2) Pharmacological studies on tanshinone I and its derivatives preliminarily revealed the development value of tanshinone I and its derivatives. Especially in the field of anti-tumor research and development, compound **135** (Figure 17) showed strong anti-cancer activity against KB and KB/VCR cells, and *in vivo* experiments, it significantly inhibited the growth of HCT116 tumor without reducing the body weight of nude mice. Utilizing the proven tanshinone I derivatives (Figure 17) and the available SAR information based on the grouped fragments that increase drug activity or improve water solubility, especially the key pharmacophore in natural medicines on the market and in clinical trials. This may provide a new strategy to develop tanshinone I derivatives with high efficacy, water solubility, bioavailability, and safety.
3) In the early stage of drug discovery, animal experiments on tanshinone I and its derivatives should be performed first because there are few animal experiments on tanshinone I and its derivatives. Modification of tanshinone I by introducing hydrophilic functions, such as **55** and **92**, can improve water solubility and anti-tumor activity. Unfortunately, these compounds have not yet achieved particularly strong pharmacological activities, and there have been no relevant *in vivo* experiments.
4) Novel drug delivery systems are effective strategies to improve water solubility, absorption, distribution, metabolism, excretion (ADME), and toxicity of many drugs (Allen and Cullis, 2013; Liu et al., 2014). A study on tanshinone I combined with a novel drug delivery system has not yet been reported. For example, conjugation of tanshinone I with receptor-selective ligands such as sugars, peptides, antibodies, and oligonucleotides, as well as the encapsulation of tanshinone I with well-designed nano-vehicles. The development of novel drug delivery formulations that include nanosuspensions, micelles, nanoparticles, and nanogels will improve the efficacy, water solubility, and targeting properties of tanshinone I formulations.
5) In most of the aforementioned studies, the reported modifications were limited to furan rings, B rings, and *o*-diquinone, while the A ring modification of this molecule is still underdeveloped. Therefore, future work should be devoted to studying A-ring modification of tanshinone I. In addition, tanshinone I derivatives have been tested in various cancer cells. Many natural products are biologically active and simultaneously provide opportunities for drug discovery in different therapeutic areas. Tanshinone I is known to have biological activities such as anti-tumor, anti-inflammatory, anti-leukemia, anti-oxidative, and neuroprotective. However, research on tanshinone I derivatives has only focused on the anti-tumor field, with the exception of compound **147**, which has antibacterial activity. We hope that researchers will explore other neuroprotective, antibacterial, and anti-inflammatory effects of tanshinone I and its derivatives.
6) The development of tanshinone I-based drug combinations is also a useful strategy, such as combining tanshinone I with other anticancer drugs to obtain high anticancer activity, thereby overcoming the limitation of insufficient anti-tumor activity of tanshinone I. We believe that tanshinone I provides a natural product platform for drug development to treat cancer and other diseases. Thus far, this platform has provided an excellent basis for the development of new derivatives that are more potent and water-soluble than the natural product tanshinone I. Tanshinone I derivatives may be potential drugs for the clinical treatment of human diseases.

## Abbreviations

LC3, light chain 3; ERK, extracellular regulated protein kinases; FDA, Food and Drug Administration; MAPK, Mitogen-activated protein kinase TNF-a tumor necrosis factor alpha; Bcl-2, B-cell lymphoma 2Bax Bcl-2-associated X protein; NF-kB, nuclear factor k light chain enhancer of activated B cells; p53, tumor protein p53; p21 p21, Cip1 protein; PI3K, phosphoinositide 3-kinase; VEGF, vascular endothelial growth factor; IL-8, Interleukin-8; IL-1β, Interleukin- 1β; COX-2, cyclooxygenase-2; AKT, Also known as PKB (protein kinase B); mTOR, Mammalian target of rapamycin; SAR, Structure Activity relationship; PGE2, prostaglandin E2; Nrf2, NF-E2-related factor.

## References

[B1] Allen T. M. Cullis P. R. (2013). Liposomal Drug Delivery Systems: From Concept to Clinical Applications. Adv. Drug Deliv. Rev. 65, 36–48. 10.1016/j.addr.2012.09.037 23036225

[B2] Baillie A. C. Thomson R. H. (1968). Naturally Occurring Quinones. Part XI. The Tanshinones. J. Chem. Soc. C. 1, 48–52. 10.1039/j39680000048

[B3] De Koning C. B. Michael J. P. Rousseau A. L. (1998). A Novel Method for the Synthesis of Phenanthrenes and Benzo[a]carbazoles. Tetrahedron Lett. 39, 8725–8728. 10.1016/s0040-4039(98)01919-4

[B4] de Oliveira M. R. Schuck P. F. Bosco S. M. D. (2017a). Tanshinone I Induces Mitochondrial Protection through an Nrf2-Dependent Mechanism in Paraquat-Treated Human Neuroblastoma SH-Sy5y Cells. Mol. Neurobiol. 54, 4597–4608. 10.1007/s12035-016-0009-x 27389776

[B5] de Oliveira M. R. Fürstenau C. R. de Souza I. C. C. da Costa Ferreira G. (2017b). Tanshinone I Attenuates the Effects of a Challenge with H2O2 on the Functions of Tricarboxylic Acid Cycle and Respiratory Chain in SH-Sy5y Cells. Mol. Neurobiol. 54, 7858–7868. 10.1007/s12035-016-0267-7 27848206

[B6] Ding C. Tian Q. Li J. Jiao M. Song S. Wang Y. (2018). Structural Modification of Natural Product Tanshinone I Leading to Discovery of Novel Nitrogen-Enriched Derivatives with Enhanced Anticancer Profile and Improved Drug-like Properties. J. Med. Chem. 61, 760–776. 10.1021/acs.jmedchem.7b01259 29294282

[B7] Dong Y. Morris-Natschke S. L. Lee K. H. (2011). Biosynthesis, Total Syntheses, and Antitumor Activity of Tanshinones and Their Analogs as Potential Therapeutic Agents. Nat. Prod. Rep. 28, 529–542. 10.1039/c0np00035c 21225077

[B8] Dun S. Gao L. (2019). Tanshinone I Attenuates Proliferation and Chemoresistance of Cervical Cancer in a KRAS-Dependent Manner. J. Biochem. Mol. Toxicol. 33, e22267. 10.1002/jbt.22267 30506648

[B9] Foulkes M. J. Tolliday F. H. Henry K. M. Renshaw S. A. Jones S. (2020). Evaluation of the Anti-Inflammatory Effects of Synthesised Tanshinone I and Isotanshinone I Analogues in Zebrafish. PLoS One 15, e0240231. 10.1371/journal.pone.0240231 33022012 PMC7537861

[B10] Gao H. Liu X. Sun W. Kang N. Liu Y. Yang S. (2017). Total Tanshinones Exhibits Anti-Inflammatory Effects Through Blocking TLR4 Dimerization via the MyD88 Pathway. Cell. Death. Dis. 8, e3004. 10.1038/cddis.2017.389 PMC5596575 28817116

[B11] Gong Y. Li Y. Lu Y. Li L. Abdolmaleky H. Blackburn G. L. (2011). Bioactive Tanshinones in Salvia Miltiorrhiza Inhibit the Growth of Prostate Cancer Cells *In Vitro* and in Mice. Int. J. Cancer 129, 1042–1052. 10.1002/ijc.25678 20848589 PMC3032031

[B12] Hao W. Chen L. Wu L. F. Yang F. Niu J. X. Kaye A. D. (2016). Tanshinone IIA Exerts an Antinociceptive Effect in Rats with Cancer-Induced Bone Pain. Pain. Physician 19, 465–476. 27676663

[B13] Huot R. Brassard P. (1974). Synthèse de méthyl-3 furoquinones. Can. J. Chem. 52, 88–94. 10.1139/v74-012

[B14] Inouye Y. Kakisawa H. (1969). Total Syntheses of Tanshinone-I, Tanshinone-II and Cryptotanshinone. Bcsj 42, 3318–3323. 10.1246/bcsj.42.3318

[B15] Jiao M. Ding C. Zhang A. (2014). Facile Construction of 3-Hydroxyphenanthrene-1,4-Diones: Key Intermediates to Tanshinone I and its A-Ring-Modified Analogue. Tetrahedron 70, 2976–2981. 10.1016/j.tet.2014.03.019

[B16] Jiao M. Ding C. Zhang A. (2015). Preparation of 2-aryl Derivatives of Tanshinone I Through a Palladium-Catalyzed Csp2-H Activation/Arylation Approach. Tetrahedron Lett. 56, 2799–2802. 10.1016/j.tetlet.2015.04.040

[B17] Jing X. Xu Y. Cheng W. Guo S. Zou Y. He L. (2016). Tanshinone I Induces Apoptosis and Pro-Survival Autophagy in Gastric Cancers. Cancer. Chemother. Pharmacol. 77, 1171–1181. 10.1007/s00280-016-3034-6 27100736

[B18] Kang B. Y. Chung S. W. Kim S. H. Ryu S. Y. Kim T. S. (2000). Inhibition of Interleukin-12 and Interferon-Gamma Production in Immune Cells by Tanshinones from Salvia Miltiorrhiza. Immunopharmacology 49, 355–361. 10.1016/s0162-3109(00)00256-3 10996033

[B19] Kim D. H. Jeon S. J. Jung J. W. Lee S. Yoon B. H. Shin B. Y. (2007). Tanshinone Congeners Improve Memory Impairments Induced by Scopolamine on Passive Avoidance Tasks in Mice. Eur. J. Pharmacol. 574, 140–147. 10.1016/j.ejphar.2007.07.042 17714702

[B20] Kim D. H. Kim S. Jeon S. J. Son K. H. Lee S. Yoon B. H. (2009). Tanshinone I Enhances Learning and Memory, and Ameliorates Memory Impairment in Mice via the Extracellular Signal-Regulated Kinase Signalling Pathway. Br. J. Pharmacol. 158, 1131–1142. 10.1111/j.1476-5381.2009.00378.x 19775283 PMC2785534

[B21] Kim D. H. Shin E. A. Kim B. Shim B. S. Kim S. H. (2018). Reactive Oxygen Species-Mediated Phosphorylation of P38 Signaling Is Critically Involved in Apoptotic Effect of Tanshinone I in Colon Cancer Cells. Phytother. Res. 32, 1975–1982. 10.1002/ptr.6126 29876988

[B22] Kim H. K. Woo E. R. Lee H. W. Park H. R. Kim H. N. Jung Y. K. (2008). The Correlation of Salvia Miltiorrhiza Extract-Induced Regulation of Osteoclastogenesis with the Amount of Components Tanshinone I, Tanshinone IIA, Cryptotanshinone, and Dihydrotanshinone. Immunopharmacol. Immunotoxicol. 30, 347–364. 10.1080/08923970801949133 18569089

[B23] Kim M. K. Park G. H. Eo H. J. Song H. M. Lee J. W. Kwon M. J. (2015). Tanshinone I Induces Cyclin D1 Proteasomal Degradation in an ERK1/2 Dependent Way in Human Colorectal Cancer Cells. Fitoterapia 101, 162–168. 10.1016/j.fitote.2015.01.010 25615593

[B24] Kim S. Y. Moon T. C. Chang H. W. Son K. H. Kang S. S. Kim H. P. (2002). Effects of Tanshinone I Isolated from Salvia Miltiorrhiza Bunge on Arachidonic Acid Metabolism and *In Vivo* Inflammatory Responses. Phytother. Res. 16, 616–620. 10.1002/ptr.941 12410540

[B25] Komodzinski K. Lepczynska J. Ruszkowski P. Milecki J. Skalski B. (2013). Biological Evaluation of an Imidazole-Fused 1,3,5-triazepinone Nucleoside and its Photochemical Generation via a 6-Azidopurine Modified Oligonucleotide. Tetrahedron. Lett. 54, 3781–3784. 10.1016/j.tetlet.2013.05.051

[B26] Lee C. Y. Sher H. F. Chen H. W. Liu C. C. Chen C. H. Lin C. S. (2008). Anticancer Effects of Tanshinone I in Human Non-Small Cell Lung Cancer. Mol. Cancer. Ther. 7, 3527–3538. 10.1158/1535-7163.MCT-07-2288 19001436

[B27] Lee J. C. Park J. H. Park O. K. Kim I. H. Yan B. C. Ahn J. H. (2013). Neuroprotective Effects of Tanshinone I from Danshen Extract in a Mouse Model of Hypoxia-Ischemia. Anat. Cell. Biol. 46, 183–190. 10.5115/acb.2013.46.3.183 24179693 PMC3811852

[B28] Lee S. Y. Choi D. Y. Woo E. R. (2005). Inhibition of Osteoclast Differentiation by Tanshinones from the Root of Salvia Miltiorrhiza Bunge. Arch. Pharm. Res. 28, 909–913. 10.1007/BF02973876 16178416

[B29] Li H. Zhang Q. Chu T. Shi H. Y. Fu H. M. Song X. R. (2012). Growth-inhibitory and Apoptosis-Inducing Effects of Tanshinones on Hematological Malignancy Cells and Their Structure-Activity Relationship. Anticancer Drugs 23, 846–855. 10.1097/CAD.0b013e328351f896 22495618

[B30] Li J. Xue Y. Fan Z. Ding C. Zhang A. (2017). Difluorination of Furonaphthoquinones. J. Org. Chem. 82, 7388–7393. 10.1021/acs.joc.7b01064 28653529

[B31] Li M.-M. Xia F. Li C.-J. Xu G. Qin H.-B. (2018). Design, Synthesis and Cytotoxicity of Nitrogen-Containing Tanshinone Derivatives. Tetrahedron Lett. 59, 46–48. 10.1016/j.tetlet.2017.11.046

[B32] Li N. Wu B. Yu C. Li T. Zhang W. X. Xi Z. (2020). Trishomoaromatic (B3 N3 Ph6 ) Dianion: Characterization and Two-Electron Reduction. Angew. Chem. Int. Ed. Engl. 59, 8868–8872. 10.1002/anie.201916651 32133711

[B33] Li X. B. Cheng X. Zhang D. L. Wu H. Q. Ye J. T. Du J. (2014). Syntheses of Tanshinone Anhydrides and Their Suppression on Oxidized LDL Uptake in Macrophages and Foam Cell Formation. Pharmazie 69, 163–167. 10.1691/ph.2014.3839 24716403

[B34] Li Y. Gong Y. Li L. Abdolmaleky H. M. Zhou J. R. (2013). Bioactive Tanshinone I Inhibits the Growth of Lung Cancer in Part via Downregulation of Aurora A Function. Mol. Carcinog. 52, 535–543. 10.1002/mc.21888 22389266 PMC3376178

[B35] Liu J. Huang Y. Kumar A. Tan A. Jin S. Mozhi A. (2014). pH-Sensitive Nano-Systems for Drug Delivery in Cancer Therapy. Biotechnol. Adv. 32, 693–710. 10.1016/j.biotechadv.2013.11.009 24309541

[B36] Liu J. Wang F. Sheng P. Xia Z. Jiang Y. Yan B. C. (2021). A Network-Based Method for Mechanistic Investigation and Neuroprotective Effect on Treatment of Tanshinone Ⅰ Against Ischemic Stroke in Mouse. J. Ethnopharmacol. 272, 113923. 10.1016/j.jep.2021.113923 33617968

[B37] Liu J. J. Liu W. D. Yang H. Z. Zhang Y. Fang Z. G. Liu P. Q. (2010a). Inactivation of PI3k/Akt Signaling Pathway and Activation of Caspase-3 Are Involved in Tanshinone I-Induced Apoptosis in Myeloid Leukemia Cells *In Vitro* . Ann. Hematol. 89, 1089–1097. 10.1007/s00277-010-0996-z 20512574

[B38] Liu X. Liu J. (2020). Tanshinone I Induces Cell Apoptosis by Reactive Oxygen Species-Mediated Endoplasmic Reticulum Stress and by Suppressing p53/DRAM-Mediated Autophagy in Human Hepatocellular Carcinoma. Artif. Cells. Nanomed. Biotechnol. 48, 488–497. 10.1080/21691401.2019.1709862 32013613

[B39] Liu X.-W. Chen Z.-Y. Wang G.-L. Ma X.-T. Gong Y. Liu X.-L. (2017). Diversity-oriented TsOH Catalysis-Enabled Construction of Tanshinone-Substituted Bis(indolyl/pyrrolyl)methanes and Their Biological Evaluation for Anticancer Activities. Synth. Commun. 47, 2378–2386. 10.1080/00397911.2017.1378359

[B40] Liu X. D. Fan R. F. Zhang Y. Yang H. Z. Fang Z. G. Guan W. B. (2010b). Down-regulation of Telomerase Activity and Activation of Caspase-3 Are Responsible for Tanshinone I-Induced Apoptosis in Monocyte Leukemia Cells *In Vitro* . Int. J. Mol. Sci. 11, 2267–2280. 10.3390/ijms11062267 20640151 PMC2904915

[B41] Liu Y. Li X. Li Y. Wang L. Xue M. (2010c). Simultaneous Determination of Danshensu, Rosmarinic Acid, Cryptotanshinone, Tanshinone IIA, Tanshinone I and Dihydrotanshinone I by Liquid Chromatographic-Mass Spectrometry and the Application to Pharmacokinetics in Rats. J. Pharm. Biomed. Anal. 53, 698–704. 10.1016/j.jpba.2010.03.041 20430561

[B42] Lu M. Wang C. Wang J. (2016). Tanshinone I Induces Human Colorectal Cancer Cell Apoptosis: the Potential Roles of Aurora A-P53 and Survivin-Mediated Signaling Pathways. Int. J. Oncol. 49, 603–610. 10.3892/ijo.2016.3565 27279458

[B43] Ma Z. L. Zhang B. J. Wang D. T. Li X. Wei J. L. Zhao B. T. (2015). Tanshinones Suppress AURKA through Up-Regulation of miR-32 Expression in Non-small Cell Lung Cancer. Oncotarget 6, 20111–20120. 10.18632/oncotarget.3933 26036635 PMC4652991

[B44] Mosaddik M. A. (2003). *In Vitro* cytotoxicity of Tanshinones Isolated from Salvia Miltiorrhiza Bunge against P388 Lymphocytic Leukemia Cells. Phytomedicine 10, 682–685. 10.1078/0944-7113-00321 14692730

[B45] Nizamutdinova I. T. Lee G. W. Lee J. S. Cho M. K. Son K. H. Jeon S. J. (2008a). Tanshinone I Suppresses Growth and Invasion of Human Breast Cancer Cells, MDA-MB-231, Through Regulation of Adhesion Molecules. Carcinogenesis 29, 1885–1892. 10.1093/carcin/bgn151 18586687

[B46] Nizamutdinova I. T. Lee G. W. Son K. H. Jeon S. J. Kang S. S. Kim Y. S. (2008b). Tanshinone I Effectively Induces Apoptosis in Estrogen Receptor-Positive (MCF-7) and Estrogen Receptor-Negative (MDA-MB-231) Breast Cancer Cells. Int. J. Oncol. 33, 485–491. 10.3892/ijo_00000031 18695877

[B47] Park E. J. Ji H. Y. Kim N. J. Song W. Y. Kim Y. H. Kim Y. C. (2008). Simultaneous Determination of Tanshinone I, Dihydrotanshinone I, Tanshinone IIA and Cryptotanshinone in Rat Plasma by Liquid Chromatography-Tandem Mass Spectrometry: Application to a Pharmacokinetic Study of a Standardized Fraction of Salvia Miltiorrhiza, PF2401-SF. Biomed. Chromatogr. 22, 548–555. 10.1002/bmc.968 18205136

[B48] Park J. H. Park O. K. Yan B. Ahn J. H. Kim I. H. Lee J. C. (2014a). Neuroprotection via Maintenance or Increase of Antioxidants and Neurotrophic Factors in Ischemic Gerbil Hippocampus Treated with Tanshinone I. Chin. Med. J. Engl. 127, 3396–3405. 25269903

[B49] Park J. H. Park O. K. Cho J.-H. Chen B. H. Kim I. H. Ahn J. H. (2014b). Anti-inflammatory Effect of Tanshinone I in Neuroprotection Against Cerebral Ischemia-Reperfusion Injury in the Gerbil Hippocampus. Neurochem. Res. 39, 1300–1312. 10.1007/s11064-014-1312-4 24760430

[B50] Pfeiffer B. Hauenstein K. Merz P. Gertsch J. Altmann K. H. (2009). Synthesis and SAR of C12-C13-Oxazoline Derivatives of Epothilone A. Bioorg. Med. Chem. Lett. 19, 3760–3763. 10.1016/j.bmcl.2009.04.112 19433359

[B51] Rajule R. Bryant V. C. Lopez H. Luo X. Natarajan A. (2012). Perturbing Pro-Survival Proteins Using Quinoxaline Derivatives: A Structure-Activity Relationship Study. Bioorg. Med. Chem. 20, 2227–2234. 10.1016/j.bmc.2012.02.022 22386982 PMC3303926

[B52] Rubottom G. M. Kim C. (1983). Preparation of Methyl Ketones by the Sequential Treatment of Carboxylic Acids with Methyllithium and Chlorotrimethylsilane. J. Org. Chem. 48, 1550–1552. 10.1021/jo00157a038

[B53] Shi M. Luo X. Ju G. Li L. Huang S. Zhang T. (2016). Enhanced Diterpene Tanshinone Accumulation and Bioactivity of Transgenic Salvia Miltiorrhiza Hairy Roots by Pathway Engineering. J. Agric. Food. Chem. 64, 2523–2530. 10.1021/acs.jafc.5b04697 26753746

[B54] Shin E. A. Sohn E. J. Won G. Choi J. U. Jeong M. Kim B. (2014). Upregulation of microRNA135a-3p and Death Receptor 5 Plays a Critical Role in Tanshinone I Sensitized Prostate Cancer Cells to TRAIL Induced Apoptosis. Oncotarget 5, 5624–5636. 10.18632/oncotarget.2152 25015549 PMC4170628

[B55] Su C. C. Chen G. W. Lin J. G. (2008). Growth Inhibition and Apoptosis Induction by Tanshinone I in Human Colon Cancer Colo 205 Cells. Int. J. Mol. Med. 22, 613–618. 10.3892/ijmm_00000063 18949381

[B56] Tao S. Justiniano R. Zhang D. D. Wondrak G. T. (2013a). The Nrf2-Inducers Tanshinone I and Dihydrotanshinone Protect Human Skin Cells and Reconstructed Human Skin against Solar Simulated UV. Redox Biol. 1, 532–541. 10.1016/j.redox.2013.10.004 24273736 PMC3836278

[B57] Tao S. Zheng Y. Lau A. Jaramillo M. C. Chau B. T. Lantz R. C. (2013b). Tanshinone I Activates the Nrf2-Dependent Antioxidant Response and Protects Against As(III)-Induced Lung Inflammation *In Vitro* and *In Vivo* . Antioxid. Redox Signal 19, 1647–1661. 10.1089/ars.2012.5117 23394605 PMC3809600

[B58] Tian X. H. Wu J. H. (2013). Tanshinone Derivatives: A Patent Review (January 2006 - September 2012). Expert Opin. Ther. Pat. 23, 19–29. 10.1517/13543776.2013.736494 23094864

[B59] Trinh H. T. Chae S. J. Joh E. H. Son K. H. Jeon S. J. Kim D. H. (2010). Tanshinones Isolated from the Rhizome of Salvia Miltiorrhiza Inhibit Passive Cutaneous Anaphylaxis Reaction in Mice. J. Ethnopharmacol. 132, 344–348. 10.1016/j.jep.2010.07.037 20732401

[B60] Tung Y. T. Chen H. L. Lee C. Y. Chou Y. C. Lee P. Y. Tsai H. C. (2013). Active Component of Danshen (Salvia Miltiorrhiza Bunge), Tanshinone I, Attenuates Lung Tumorigenesis via Inhibitions of VEGF, Cyclin A, and Cyclin B Expressions. Evid. Based. Complement. Altern. Med. 2013, 319247. 10.1155/2013/319247 PMC3638627 23662128

[B61] Wang D. Xu D. Sun Y. Wu Y. Ma L. Duan J. (2017a). Synthesis, Characterization and Anticancer Activity of Tanshinone I Grafted Low Molecular Chitosan. Glycoconj J. 34, 3–12. 10.1007/s10719-016-9712-0 27627976

[B62] Wang D. Lu C. Sun F. Cui M. Mu H. Duan J. (2017b). A Tanshinone I Derivative Enhances the Activities of Antibiotics against *Staphylococcus aureus In Vitro* and *In Vivo* . Res. Microbiol. 168, 46–54. 10.1016/j.resmic.2016.08.002 27545500

[B63] Wang D. Zhang W. Wang T. Li N. Mu H. Zhang J. (2015a). Unveiling the Mode of Action of Two Antibacterial Tanshinone Derivatives. Int. J. Mol. Sci. 16, 17668–17681. 10.3390/ijms160817668 26263982 PMC4581214

[B64] Wang F. Yang H. Yu S. Xue Y. Fan Z. Liang G. (2018). Total Synthesis of (±)-Tanshinol B, Tanshinone I, and (±)-Tanshindiol B and C. Org. Biomol. Chem. 16, 3376–3381. 10.1039/c8ob00567b 29670981

[B65] Wang L. Wu J. Lu J. Ma R. Sun D. Tang J. (2015b). Regulation of the Cell Cycle and PI3K/Akt/mTOR Signaling Pathway by Tanshinone I in Human Breast Cancer Cell Lines. Mol. Med. Rep. 11, 931–939. 10.3892/mmr.2014.2819 25355053 PMC4262478

[B66] Wang S. Jing H. Yang H. Liu Z. Guo H. Chai L. (2015c). Tanshinone I Selectively Suppresses Pro-Inflammatory Genes Expression in Activated Microglia and Prevents Nigrostriatal Dopaminergic Neurodegeneration in a Mouse Model of Parkinson's Disease. J. Ethnopharmacol. 164, 247–255. 10.1016/j.jep.2015.01.042 25666429

[B67] Wang W. Li J. Ding Z. Li Y. Wang J. Chen S. (2019a). Tanshinone I Inhibits the Growth and Metastasis of Osteosarcoma via Suppressing JAK/STAT3 Signalling Pathway. J. Cell. Mol. Med. 23, 6454–6465. 10.1111/jcmm.14539 31293090 PMC6714145

[B68] Wang X. Fan J. Ding X. Sun Y. Cui Z. Liu W. (2019b). Tanshinone I Inhibits IL-1β-Induced Apoptosis, Inflammation and Extracellular Matrix Degradation in Chondrocytes CHON-001 Cells and Attenuates Murine Osteoarthritis. Drug Des. Devel Ther. 13, 3559–3568. 10.2147/DDDT.S216596 PMC6800556 31686786

[B69] Wang X. Morris-Natschke S. L. Lee K. H. (2007). New Developments in the Chemistry and Biology of the Bioactive Constituents of Tanshen. Med. Res. Rev. 27, 133–148. 10.1002/med.20077 16888751

[B70] Wang Y. Li J. X. Wang Y. Q. Miao Z. H. Wang Y. (2015d). Tanshinone I Inhibits Tumor Angiogenesis by Reducing Stat3 Phosphorylation at Tyr705 and Hypoxia-Induced HIF-1α Accumulation in Both Endothelial and Tumor Cells. Oncotarget 6, 16031–16042. 10.18632/oncotarget.3648 26202747 PMC4599254

[B71] Wang Z. Y. Liu J. G. Li H. Yang H. M. (2016). Pharmacological Effects of Active Components of Chinese Herbal Medicine in the Treatment of Alzheimer's Disease: A Review. Am. J. Chin. Med. 44, 1525–1541. 10.1142/S0192415X16500853 27848250

[B72] Wei Y. Gao J. Qin L. Xu Y. Wang D. Shi H. (2017). Tanshinone I Alleviates Insulin Resistance in Type 2 Diabetes Mellitus Rats Through IRS-1 Pathway. Biomed. Pharmacother. 93, 352–358. 10.1016/j.biopha.2017.06.040 28651236

[B73] Wu L. Yang X. (2014). Synthesis and Cytotoxic Activity of Tanshinone I Derivatives Having Azacyclo Moiety. J. Chem. Pharm. Res. 6, 442–445.

[B74] Wu N. Ma W. C. Mao S. J. Wu Y. Jin H. (2017). Total Synthesis of Tanshinone I. J. Nat. Prod. 80, 1697–1700. 10.1021/acs.jnatprod.7b00238 28443671

[B75] Xu L. Feng J. M. Li J. X. Zhu J. M. Song S. S. Tong L. J. (2013). Tanshinone-1 Induces Tumor Cell Killing, Enhanced by Inhibition of Secondary Activation of Signaling Networks. Cell. death. Dis. 4, e905. 10.1038/cddis.2013.443 24201804 PMC3847321

[B76] Yang H. R. Wang J. J. Shao P. P. Yuan S. Y. Li X. Q. (2016). A Facile Three-Step Total Synthesis of Tanshinone I. J. Asian. Nat. Prod. Res. 18, 677–683. 10.1080/10286020.2015.1136906 26828227

[B77] Yu H. Subedi R. K. Nepal P. R. Kim Y. G. Choi H. K. (2012). Enhancement of Solubility and Dissolution Rate of Cryptotanshinone, Tanshinone I and Tanshinone IIA Extracted from Salvia Miltiorrhiza. Arch. Pharm. Res. 35, 1457–1464. 10.1007/s12272-012-0816-1 22941489

[B78] Zhang D. L. Zhou L. Y. Quan J. M. Zhang W. Gu L. Q. Huang Z. S. (2013). Oxygen Insertion of O-Quinone Under Catalytic Hydrogenation Conditions. Org. Lett. 15, 1162–1165. 10.1021/ol400164e 23452325

[B79] Zhou J. Jiang Y. Y. Chen H. Wu Y. C. Zhang L. (2020). Tanshinone I Attenuates the Malignant Biological Properties of Ovarian Cancer by Inducing Apoptosis and Autophagy via the Inactivation of PI3K/AKT/mTOR Pathway. Cell. Prolif. 53, e12739. 10.1111/cpr.12739 31820522 PMC7046305

[B80] Zhou W. Huang Q. Wu X. Zhou Z. Ding M. Shi M. (2017). Comprehensive Transcriptome Profiling of Salvia Miltiorrhiza for Discovery of Genes Associated with the Biosynthesis of Tanshinones and Phenolic Acids. Sci. Rep. 7, 10554–10612. 10.1038/s41598-017-10215-2 28874707 PMC5585387

[B81] Zhou Y. Li W. Xu L. Chen L. (2011). In Salvia Miltiorrhiza, Phenolic Acids Possess Protective Properties Against Amyloid β-Induced Cytotoxicity, and Tanshinones Act as Acetylcholinesterase Inhibitors. Environ. Toxicol. Pharmacol. 31, 443–452. 10.1016/j.etap.2011.02.006 21787715

